# Cell-Specific Cardiac Electrophysiology Models

**DOI:** 10.1371/journal.pcbi.1004242

**Published:** 2015-04-30

**Authors:** Willemijn Groenendaal, Francis A. Ortega, Armen R. Kherlopian, Andrew C. Zygmunt, Trine Krogh-Madsen, David J. Christini

**Affiliations:** 1 Greenberg Division of Cardiology, Weill Cornell Medical College, New York, New York, United States of America; 2 Department of Physiology and Biophysics, Weill Cornell Medical College, New York, New York, United States of America; 3 Consultant, New Hartford, New York, United States of America; 4 Institute for Computational Biomedicine, Weill Cornell Medical College, New York, New York, United States of America; University of California San Diego, UNITED STATES

## Abstract

The traditional cardiac model-building paradigm involves constructing a composite model using data collected from many cells. Equations are derived for each relevant cellular component (e.g., ion channel, exchanger) independently. After the equations for all components are combined to form the composite model, a subset of parameters is tuned, often arbitrarily and by hand, until the model output matches a target objective, such as an action potential. Unfortunately, such models often fail to accurately simulate behavior that is dynamically dissimilar (e.g., arrhythmia) to the simple target objective to which the model was fit. In this study, we develop a new approach in which data are collected via a series of complex electrophysiology protocols from single cardiac myocytes and then used to tune model parameters via a parallel fitting method known as a genetic algorithm (GA). The dynamical complexity of the electrophysiological data, which can only be fit by an automated method such as a GA, leads to more accurately parameterized models that can simulate rich cardiac dynamics. The feasibility of the method is first validated computationally, after which it is used to develop models of isolated guinea pig ventricular myocytes that simulate the electrophysiological dynamics significantly better than does a standard guinea pig model. In addition to improving model fidelity generally, this approach can be used to generate a cell-specific model. By so doing, the approach may be useful in applications ranging from studying the implications of cell-to-cell variability to the prediction of intersubject differences in response to pharmacological treatment.

## Author Summary

Mathematical models of cardiac cell electrophysiology are widely used as predictive and illuminatory tools, but have been developed for decades using a suboptimal process. The models are typically constructed by manual adjustment of parameters to fit simple data and therefore often underperform when used to predict complex behavior such as arrhythmias. We present a novel method of model parameterization using automated optimization and dynamically rich fitting data and then demonstrate that this approach is better at finding the “real” model of a cell. Application of the method to cardiac myocytes leads to cell-specific models, which may enable well-controlled studies of both cellular- and subject-level population heterogeneity in disease propensity and response to therapies.

## Introduction

Mathematical models of cardiac electrophysiology trace their roots to Hodgkin and Huxley’s seminal work from 1952 [1]. Since then many models have been developed describing cardiac electrophysiology in a number of species and cell types helping to make modeling an integral part of cardiac research [2–5].

The typical method for model development and parameterization is a bottom-up approach. Individual ionic membrane currents are characterized using voltage-clamp experiments from which mathematical equations are derived [6,7]. Although it has led to many advances, this traditional approach to model development has several limitations, including:

1. Because individual quantification of all membrane currents requires many experiments, model-development data are typically taken from multiple laboratories, often using different experimental protocols with varying conditions such as temperature and solution composition that directly influence the ionic currents [7,8]. Such variations may be taken into account by the modeler, but extrapolations to other conditions are often based on sporadic data [9].
2. Data may originate from cells from different locations within the heart, or even different species, which can be problematic because of regional/species heterogeneities in electrophysiological properties [8]. The data may also come from animals or patients with divergent characteristics, such as gender and age, that influence electrical activity.
3. An additional cause of inconsistency comes from averaging over multiple experiments of both the voltage clamp data used to build the model and the functional data used to parameterize it. Even in myocytes from the same region of the heart, there is considerable cell-to-cell variability in the action potential, presumably stemming from different levels of ion channel densities. Thus, models create the ‘average’ behavior for a cardiac myocyte, although no single cell necessarily behaves like the average model [10,11].
4. Another problematic issue arises when the data and equations for the individual currents are combined to build a composite model. This step typically requires tuning of model parameters to reproduce whole-cell behavior; this tuning is usually done manually in a laborious, iterative tweaking process, which ends when the model output (e.g., an action potential) subjectively is deemed to adequately match the experimental counterparts. Such manual adjustments will almost certainly not result in the best possible fit to the data, as the approach can explore only a small fraction of parameter space.
5. Given the relatively simple dynamics (e.g., often a single action potential) to which the models are tuned, models often fare poorly when attempting to simulate more complex behaviors (e.g., fibrillatory dynamics, drug block, or pacing interventions) [7,12–14]. This is unfortunate as one of the key goals of cardiac modeling is to predict and give mechanistic insights into arrhythmias, which often occur at excitation patterns different from those used to construct the models.

Several studies have looked into how to improve model parameterization. Approaches in cardiac myocyte modeling have included the parameterization of individual channel dynamics, typically when making more complex Markov models of ionic currents [6,15–17]. Whole-cell optimization approaches have focused on generating models that can match action potentials from different types of cardiomyocytes, using both simple models consisting of a few generic currents [13,18–20] and more physiologically detailed ionic models [21–25]. A resulting synthesis is that optimization results are improved when models are fit to data beyond a single action potential, e.g., action potentials from multiple pacing rates [13,19,22] or voltage waveforms during varying current injection [20,21,24]. In particular, using a global search heuristic applied to an ionic model, Syed et al. demonstrated that it is feasible to estimate conductance parameters for experimental data and showed that the fits improved when using data recorded during multiple periodic pacing frequencies [22]. Sarkar and Sobie presented a much simpler, but more approximate, linear regression strategy to estimate model conductances based on biomarkers from simulated model output and have used it to investigate how specific conductances relate to particular model outputs [26]. In neuroscience, considerably more research has been carried out on parameter estimation problems (e.g., [27–30]) and a few studies have developed protocols that allow parameterization of cell-specific models [31,32]. However, these protocols are not directly applicable to cardiac myocytes, due to intrinsic differences in electrophysiological behavior between neurons and cardiomyocytes.

Here, we present a novel strategy to develop cardiac models by pairing dynamically rich electrophysiology protocols with powerful computational parameter fitting methods. We first developed novel electrophysiology protocols that probe the dynamics of a subset of ionic currents in an intact cardiac myocyte without ion channel inhibitors, agonists, or unphysiological ion concentrations. The protocol consists of (1) stochastic current-clamp stimulation and (2) multi-step voltage clamping. As will be discussed, stochastic stimulation represents a quick method to sample the rate-dependent cardiac dynamics, while the multi-step voltage-clamp protocol is designed to highlight individual currents using a tailored sequence of holding potentials. Based on the assumption that ion-channel kinetics are preserved among (healthy) subjects while maximal conductances vary as a result of differences in expression levels, the resulting data are used to estimate maximal conductance values of several ionic currents and maximal flux of calcium ion transporters in the model. Because of the complexity of the data, hand parameter tuning is not feasible; thus, we utilized a genetic algorithm (GA), which is an efficient method for such complex optimization applications [33]. The approach was first developed and validated computationally. It was then used to develop cell-specific models of isolated guinea pig ventricular myocytes that simulate the electrophysiological dynamics significantly better than does a standard guinea pig model.

## Results

### GA optimization using a single action potential

We first developed our parameter estimation strategy using a guinea pig ventricular myocyte model (Faber and Rudy [34], the “FR” model) and tested the ability of the optimization procedure to return the original parameter values. Traditionally, one of the main criteria for cardiac electrophysiology model quality is the ability of a model to describe the cardiac action potential. Therefore, we first ran the parameter estimation using a single FR model action potential as the target objective. Nine model parameters, describing maximal conductances of ionic currents [the sodium current (I_Na_), the L-type calcium current (I_CaL_), the T-type calcium current (I_CaT_), the inwardly rectifying potassium (I_K1_), the rapid and slow delayed rectifier potassium currents (I_Kr_ and I_Ks_), the plateau potassium current (I_Kp_), and the sarcolemmal calcium pump current (I_pCa_)] and the maximal flux of the sarcoplasmic reticulum Ca^2+^-ATPase (J_SERCA_) were estimated using a GA technique.

**Fig 1:**
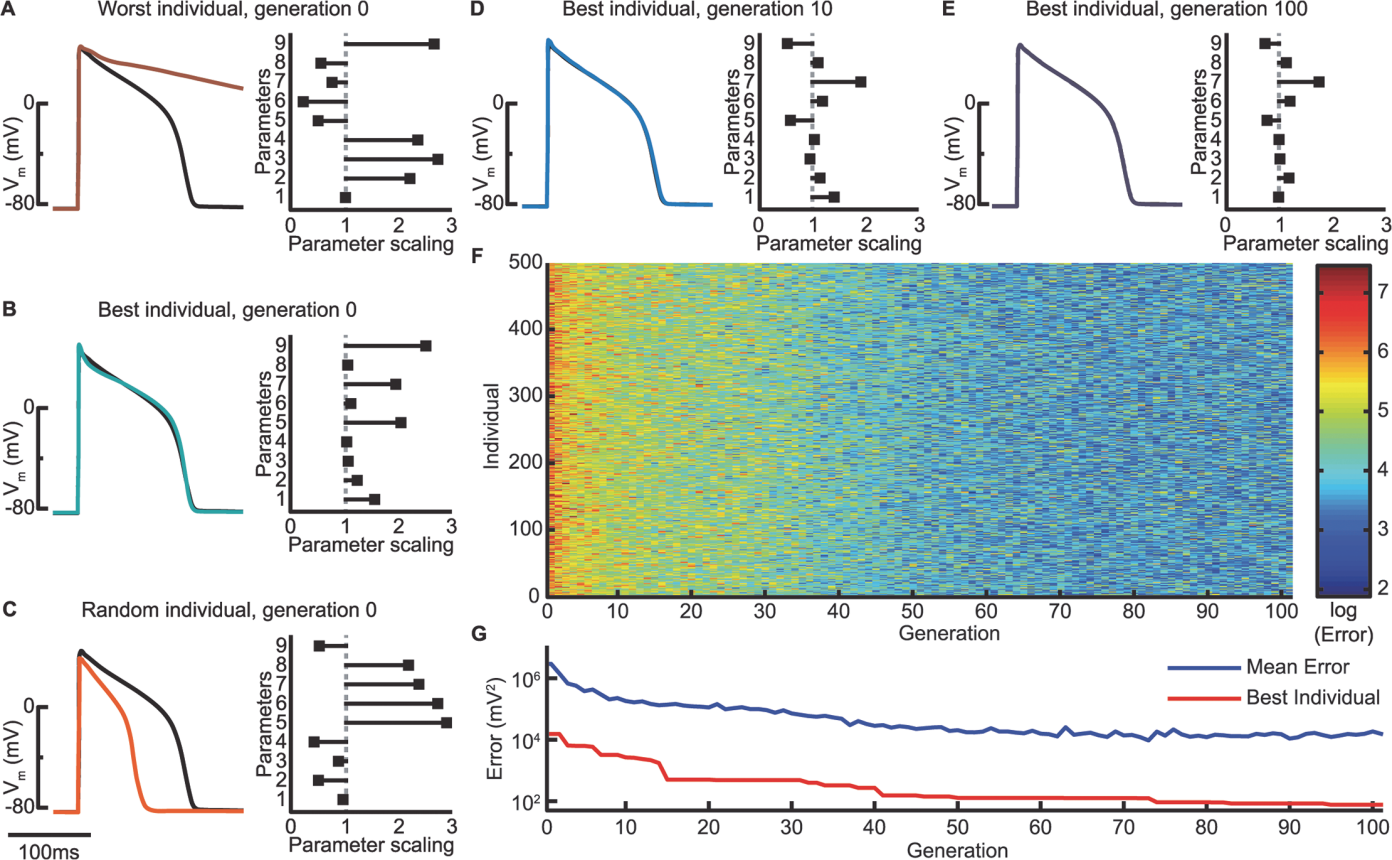
Progression of GA estimation. (A-C) The GA is initialized with 500 random individuals, i.e., model instantiations, in generation 0. Each individual is simulated according to the protocol, here a single action potential, and its error is calculated (sum of squared errors between model output and FR model target objective; Eq 2 in the Methods). Left columns show action potentials generated by three different generation 0 model instantations (traces are colored according to their error and color bar in panel F) compared to the baseline FR model (black). Right column bar graphs indicate the scaling of the nine model parameters for each individual, with a scaling of 1 representing the original FR model value. Parameters 1 through 9 correspond to: I_Na_, I_CaL_, I_CaT_, I_K1_, I_Kr_, I_Ks_, I_Kp_, I_pCa_, and J_SERCA_, respectively. (D-G) With progression through the generations, individual action potentials become more similar to the optimization objective and errors decrease accordingly. At generation 100, the overall best individual and the FR model appear very similar, although the bar graph indicates differences among the parameters (E).

A GA optimization is initialized by a population of models with different parameter values. We used a population size of 500 model instantiations generated by randomly drawing values for the nine parameters from a range of 0.01–299% of the published value. The GA methodology uses ideas from evolution [23,33]. In GA terminology, the initial state is referred to as generation 0 and the 500 models as individuals. Fig 1 A–1 C shows three different individuals in generation 0. Most of these generate action potentials that are very different from the target action potential (Fig 1A and 1C). However, by chance, a few of the 500 individuals provide reasonably good fits to the action potential, even though the parameter values are very different from those of the baseline model (parameter scaling of 1, Fig 1B).

The optimization proceeds in generations (steps), for which the GA applies crossover (parameter swapping), mutation (parameter variation), and selection (discarding poorly performing models) to increase the fitness of the population (reduce the error between model output and target objective). We ran the GA for 100 generations, as this was sufficient for the error of both the population average and the best individual to reach a minimum (Fig 1 F and 1G). Although the optimized model action potential matches the optimization objective to a very high degree, the estimated parameter set does not match that of the FR model (Fig 1E). This is consistent with previous results showing that if only a single action potential is used for parameterization, cardiac models may be overdetermined and more than a single set of parameter values can describe that action potential [20,21,26].

### Stochastic stimulation protocol improves model fit and predictability over single action potential protocol

**Fig 2:**
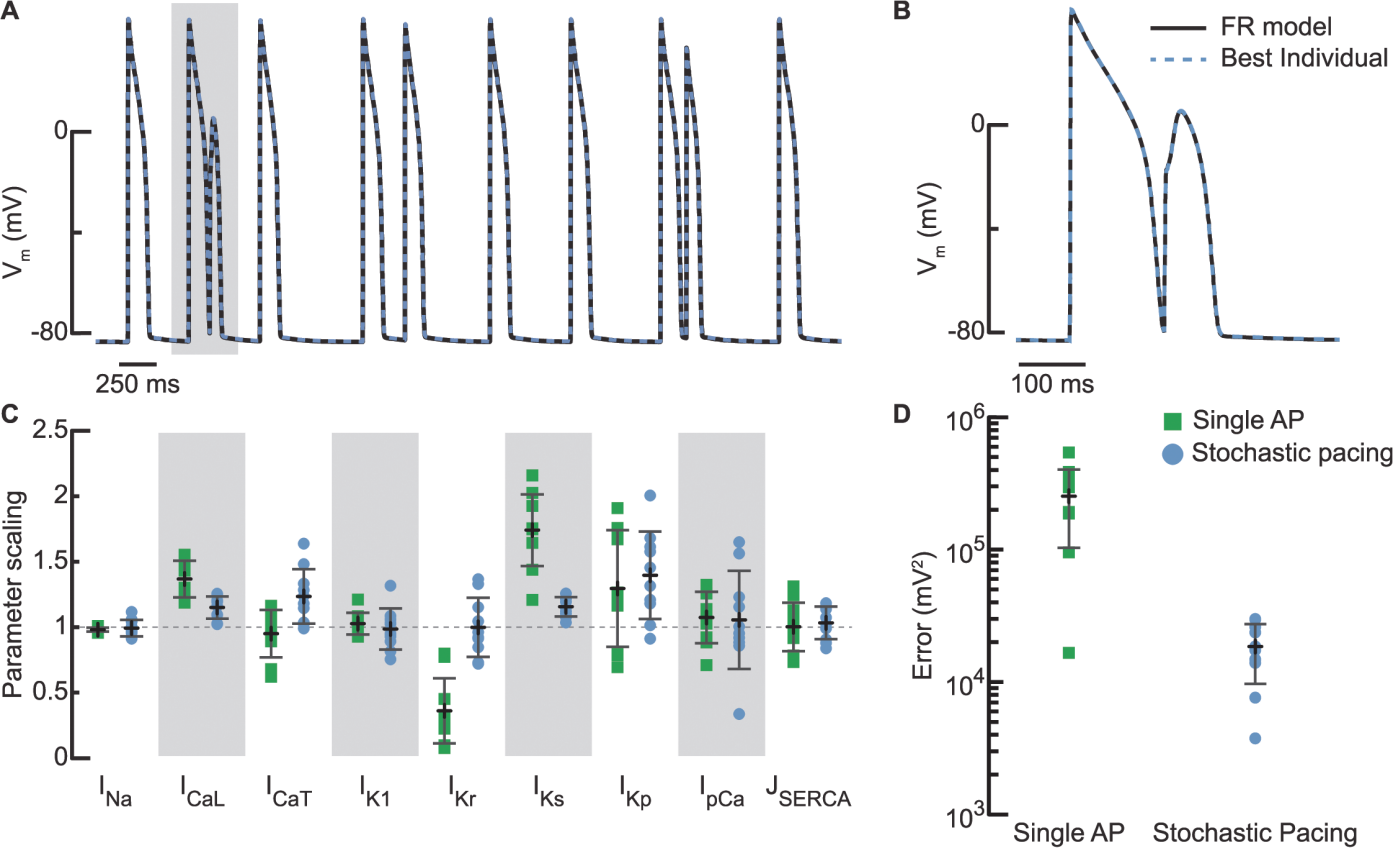
Stochastic stimulation improves parameter estimation and predictability. (A) The stochastic stimulation optimization sequence (black) and the best fit from 10 GA runs applied to fit it (light blue dashed line) show the quality of the GA fit. (B) Zoom-in of shaded region in (A) shows fitness of closely coupled action potentials. (C) Using the stochastic stimulation optimization sequence in the objective function causes some improvement in parameter recovery (light blue circles) compared to using a single action potential (green squares), most notably for I_Kr_, I_CaL_, and I_Ks_ conductances. Symbols indicate the best individual from each of the 10 individual GA runs, error bars give the mean (black) ± standard deviation (gray), and the dashed line at parameter scaling 1 indicates the original FR model parameter values. (D) When subjected to a novel stimulation sequence, the error between the model fit based on the stochastic stimulation sequence and the FR model (light blue circles) is significantly smaller (p-value of 0.0001) than the error between FR model and the best fit based on a single action potential only (green squares), i.e., the stochastic stimulation objective leads to models with higher prediction accuracy.

The duration of the action potential, and to a lesser extent its morphology, varies with stimulation interval and history. Models tuned to single action potentials during periodic pacing (as in Fig 1) would not be expected to accurately reproduce such dynamics. A more complete method to probe cellular behavior is the restitution portrait [35], which is a systematic, but prohibitively long, mapping of this rate dependence. An alternative approach that would significantly reduce the protocol duration, while maintaining some dynamic information, is random sampling of the rate dependence. To accomplish this, we utilized a stochastic stimulation protocol containing 11 randomly timed stimuli delivered over 5 s. When applied to the FR model, the stochastic stimulation results in considerable action potential variability (Fig 2 A and 2B).

We used this stochastic stimulation protocol and resulting voltage response as an optimization sequence to test the extent to which dynamic stimulus timing would improve the parameter estimation. Because the GA parameter estimation is a stochastic method, it was run 10 times with 10 different initial populations. For each run, we selected the best individual, i.e., the model instantiation with the best fit to the target voltage trace. All 10 best individuals matched the voltage trace very closely (Fig 2 A and 2B show the best one) as was the case for the single action potential fitting. Compared to the single action potential, the stochastic stimulation leads to a modest overall improvement of the parameter estimation, but it did notably better in determining the maximal conductances of I_Kr_, I_CaL_, and I_Ks_ (Fig 2C). However, as shown in Fig 2C, a few parameters remain incorrectly estimated (maximal conductances of I_CaL_ and I_Ks_), and some are estimated with a large spread (maximal conductance values of I_Kp_, I_pCa_, I_CaT_, and I_Kr_). Sensitivity and correlation analyses illuminate why these parameters are less well determined—they have low sensitivity (in which case they have minimal effect on the fitting objective, making them difficult to probe) and/or they have inter-correlations (in which case two or more parameters lack independent contributions to the fitting objective and are therefore difficult to discriminate) (S1–S4 Figs and S1 Text).

To measure the predictive capabilities of the optimized models, we presented the 10 best individuals from the GA runs with a novel stochastic stimulation sequence (S6 Fig). When subjected to this new prediction sequence, both the individuals trained with the stochastic stimulation sequence and those trained to the single action potential matched the baseline FR model response well (S6 Fig), but the stochastic stimulation protocol lead to the better match, as shown by the smaller prediction error (error between optimized model and target during the prediction sequence) in Fig 2D. Thus, although the parameter recovery seem only modestly improved for the stochastic stimulation compared to the single action potential target, the stochastic stimulation outperforms the single action potential in that it results in models that are significantly better at predicting the response to a novel set of stimuli.

### Extending the protocol: Adding multi-step voltage clamp data

To improve parameter estimation accuracy, more improvement is typically gained from adding measurements of a different state variable than adding additional measurements of the same state variable [36]. This suggests that a longer stochastic stimulation protocol is unlikely to yield much improvement. This idea is in line with our finding above (Figs 2D and S6) that models optimized to the 5s stochastic stimulation protocol matched well when subjected to a novel stochastic stimulation sequence.

**Fig 3:**
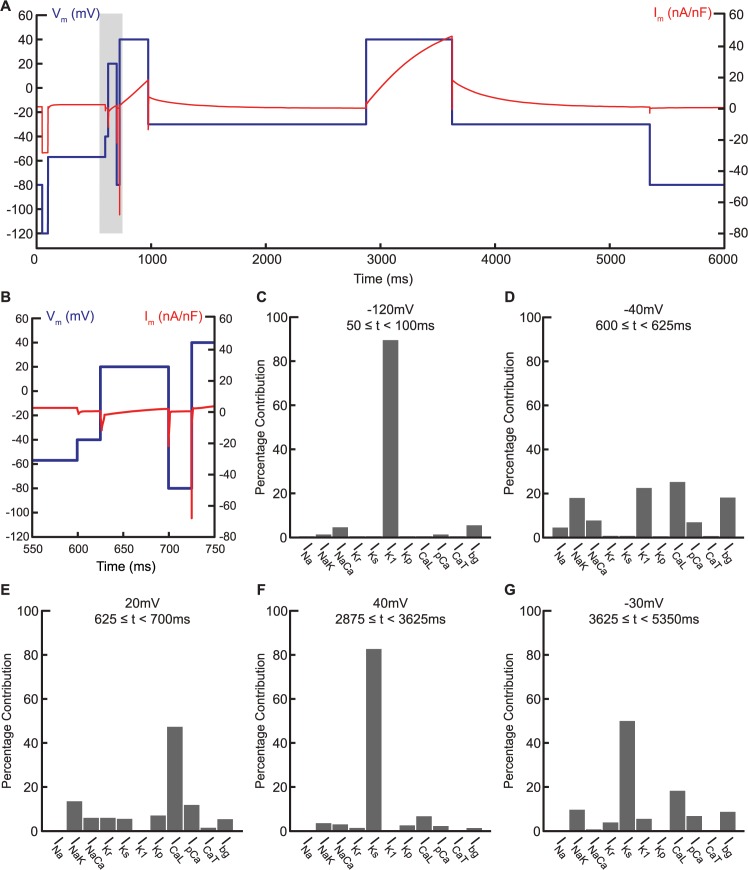
Multi-step voltage clamp protocol. (A) Voltage clamp protocol (blue) and current response (red). (B) Close-up of shaded region in (A) where voltage steps change rapidly. (C-G) Percentage contributions of the individual currents during particular parts of the protocol isolates I_K1_ (C), I_CaL_ (E) and I_Ks_ (F and G) well.

Therefore, to improve the parameter estimation accuracy, we added a multi-step voltage clamp protocol to the objective function. Traditionally, voltage clamp is applied to a cell as a set of holding potentials varied systematically either in its timing or its potential to characterize one particular current. We developed a voltage clamp protocol consisting of a sequence of holding potentials, with each step designed to emphasize specific currents relative to the others (Fig 3). The rationale is that if a particular conductance contributes most of the total current for a particular holding potential, then only models fit with a correct value of that conductance will reproduce the current target for that phase of the protocol and therefore have a low fitting error. Our 6s long voltage clamp protocol effectively isolates I_K1_, I_CaL_, and I_Ks_ as shown by the disproportionally large contributions of these currents in step -120 mV, +20 mV, and +40 and -30 mV, respectively (Fig 3 B–3F). Therefore, we hypothesize that this protocol will directly improve the conductance estimation for these currents.

### The combined stochastic current and multi-step voltage clamp protocol improves parameter estimation

**Fig 4:**
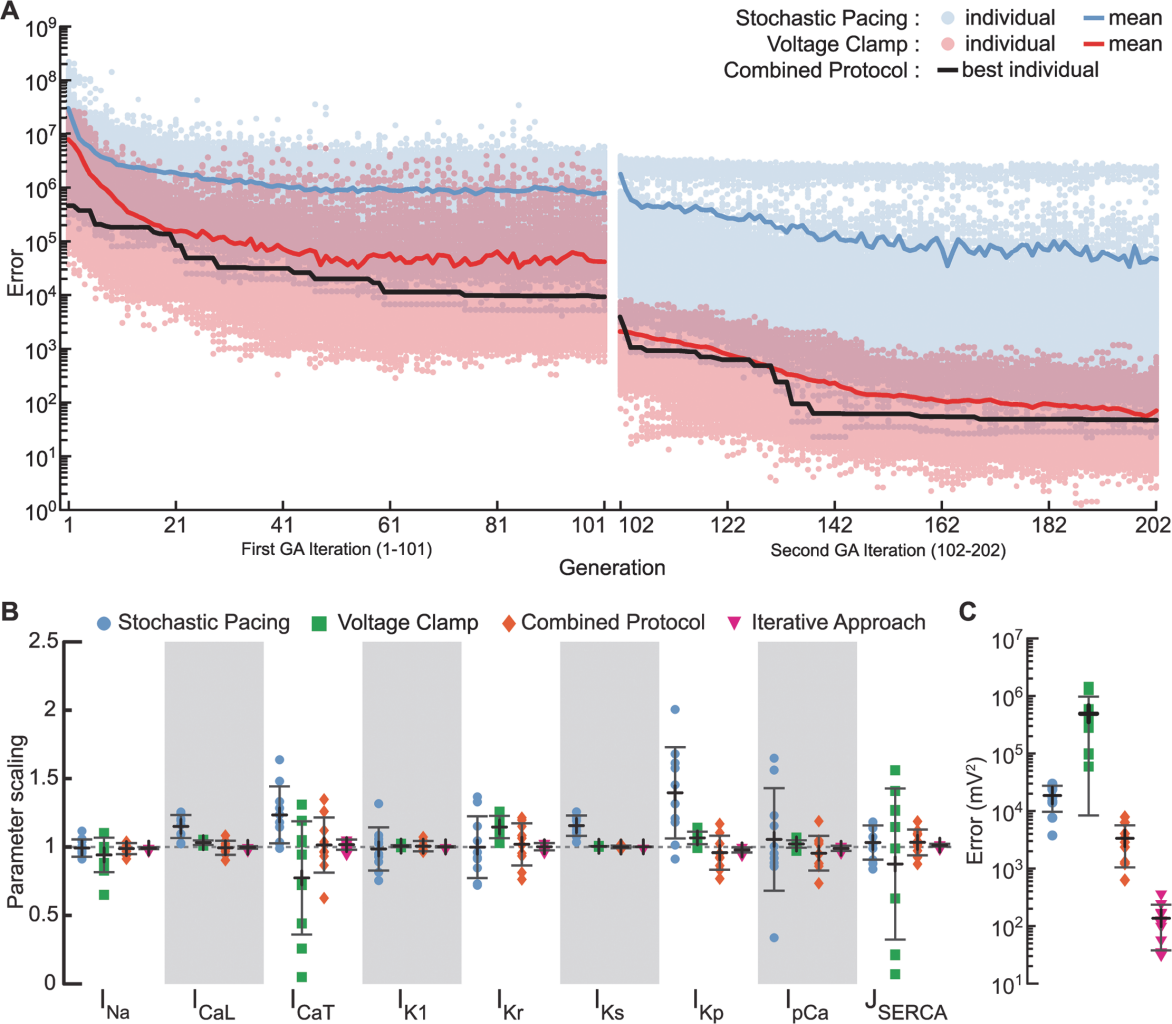
Improvement of the optimization results with inclusion of voltage-clamp protocol and use of iterative optimization technique. (A) Error reduction during optimization with combined stochastic pacing and voltage clamp objective. Circles indicate contribution to the summed error from each of the 500 individuals during stochastic pacing (blue) and voltage clamp (red), while the correspondingly colored traces give the population means. The summed error of the best individual is shown as the black trace. (B) Improvements in the parameter estimation from using stochastic stimulation (light blue circles) or voltage clamping (green squares), to using the combined stochastic pacing and voltage clamp protocol (orange diamonds), and to using the iterative optimization (magenta triangles) as visualized with estimation results centered around 1 and tighter error bars. Symbols indicate results from the individual runs; error bars give the mean (black) ± standard deviation (gray). (C) The prediction error is large for the voltage clamp protocol alone (green squares), which does not train models according to membrane potential. Adding the voltage clamp protocol to the stochastic pacing protocol (i.e., combined protocol; orange diamonds) gives better predictions compared to stochastic stimulation alone (light blue circles; p = 0.00005). The prediction error is further reduced when using the iterative optimization approach (magenta triangles; p = 0.0003).

Indeed, using the voltage clamp protocol as the objective during an optimization recovers the conductances for I_K1_ and I_Ks_ very accurately (Fig 4B). The estimation of the I_CaL_ conductance is very close to 1, but is slightly overestimated in all runs. Less predictively, I_pCa_ and I_Kp_ were also estimated more precisely than during stochastic pacing alone (Fig 4B). However, a few conductances were estimated poorly (in particular J_SERCA_ and I_CaT_) and models optimized based on voltage clamp data alone were, not surprisingly, inferior at predicting complex action potential dynamics during stochastic pacing (Fig 4C).

The extension of the target objective to include the multi-step voltage clamp protocol results in a joined unitless error function (Eq 3 in the Methods). As both the stochastic pacing recording and the voltage clamp data is fit increasingly well during optimization, the error contribution from each sequence decreases (Fig 4A, left). Although the main contribution to the total error comes from the stochastic pacing segment, the error from voltage clamp segment drops more during the optimization process, suggesting that both protocols help constrain the parameters.

Running the optimization with the combined objective does indeed lead to improved accuracy of the parameter estimation, with all nine current parameters being recovered to within one standard deviation (orange symbols, Fig 4B). For six of the nine current parameters, the combined protocol results in parameters whose mean estimates are closer to 1 and/or have less variational spread than either of the individual protocols alone (Fig 4B). Only currents that were estimated very accurately by the voltage clamp protocol alone (I_Ks_, I_K1_, and I_pCa_) did not show improvement with the combined protocol.

For some of the currents, one protocol segment is clearly better than the other in terms of parameter recovery (e.g., stochastic pacing for I_Na_ and voltage clamp for I_Ks_). However, for other currents, in which both individual protocol segments result in off-target outcomes, the combined protocol produces estimates spanning 1 (e.g., I_Kp_). Such improvement is consistent with the combined protocol restraining parameter space and avoiding local minima.

Again, we tested the ability of the 10 best individuals to predict the response to a stochastic stimulation sequence to which they were not fit. The prediction error of the 10 individuals from the combined objective function runs was significantly lower than the error for the individuals that were estimated using only the stochastic stimulation protocol (Fig 4C). Hence, the combined stochastic pacing and voltage clamp protocol improves both parameter recovery and prediction performance.

To further improve the estimation results, the results of the first 10 GA runs were used as the new parameter bounds for a second set of runs (see Methods), e.g. 0.01% to 299% changed to 92.8–114.9% for I_Na_. Note that this method only works when the fits of the first 10 runs span the correct solution as is the case with the combined protocol. During this second, local, iteration, better fits are generated causing the error for both the voltage clamp and the current segments, as well as the total error for the best individual, to drop (Fig 4A, right). Thus, using this iterative approach, the error bounds around the estimated parameter values decreased (magenta symbols, Fig 4B) and the prediction error reduced markedly (Fig 4C).

In summary, the combined protocol, consisting of stochastic stimulation and multi-step voltage clamp, allows accurate parallel estimation of eight maximal conductance values and maximal pump rate of SERCA for the FR guinea pig ventricular model. Such validation simulations laid the groundwork for using the method to fit computational models to real cardiac cell data.

### Dynamic electrophysiology protocols and optimization improve model fit to *in vitro* experimental data

The parameter estimation method was next applied to four guinea pig left basal ventricular myocytes from four different animals. Each cell was subjected to the stochastic stimulation protocol in current clamp mode, followed by the multi-step voltage clamp protocol, using the perforated patch clamp technique.

**Fig 5:**
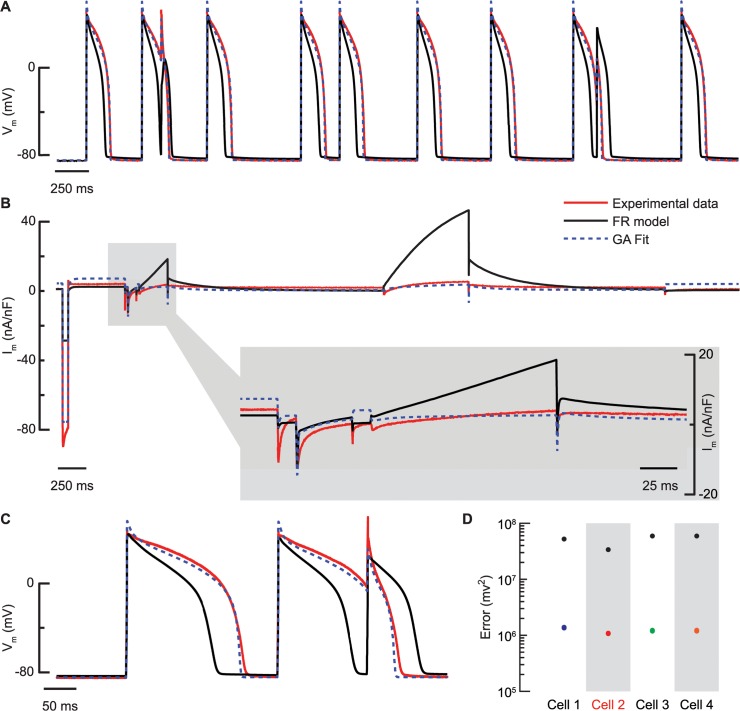
GA voltage and current recovery for in vitro myocyte compared to FR model. (A) Stochastic stimulation optimization sequence shows the difference between the experimental voltage response (red trace) and the original FR model (black trace). The GA fitted model (blue, dashed trace) matches the experimental action potentials closely. (B) The multi-step voltage clamp protocol reveals that the experimental current data is also very different from the original FR model, in particular during the first I_K1_-inducing step which results in a much larger current in the experimental recording, and during I_Ks_-inducing steps which trigger much larger currents in the model. Again, the experimental data is well fit by the optimized model. (C) The optimized model also predicts the response to a novel stochastic stimulation sequence well. This is reflected in the prediction error (D), which is reduced for the optimized models for all four cells (colored circles; standard deviation of the 10 best individuals for each cell falls with the circles) compared to the FR model (black circles). The data shown in (A-C) is from Cell 2. In (A) and (B) stimulus artifacts and capacitative currents were removed (see Methods), but data sets were plotted as continuous traces to ease visualization.

All four myocytes exhibited action potentials and membrane current responses that were very different from the baseline FR model (Fig 5 shows output from one cell, S7–S9 Figs presents the results from the remaining three cells). In particular, their action potentials were substantially longer than those of the FR model and their current response to prolonged depolarization was substantially smaller.

For each cell, the GA estimate from the experimental data fit much better than did the FR model (Fig 5 and S7–S9 Figs). In particular, the optimization leads to very accurate voltage dynamics, which is important for arrhythmogenesis prediction. The total current is fit less well, potentially due to mismatch in ion channel kinetics (see Discussion). Overall, the optimization results in more accurate predictions, with the prediction error being an order of magnitude lower for the fitted models than for the FR model (Fig 5C and 5D).

### Parameter estimation shows changes compared to FR model and variability among individual cells

**Fig 6:**
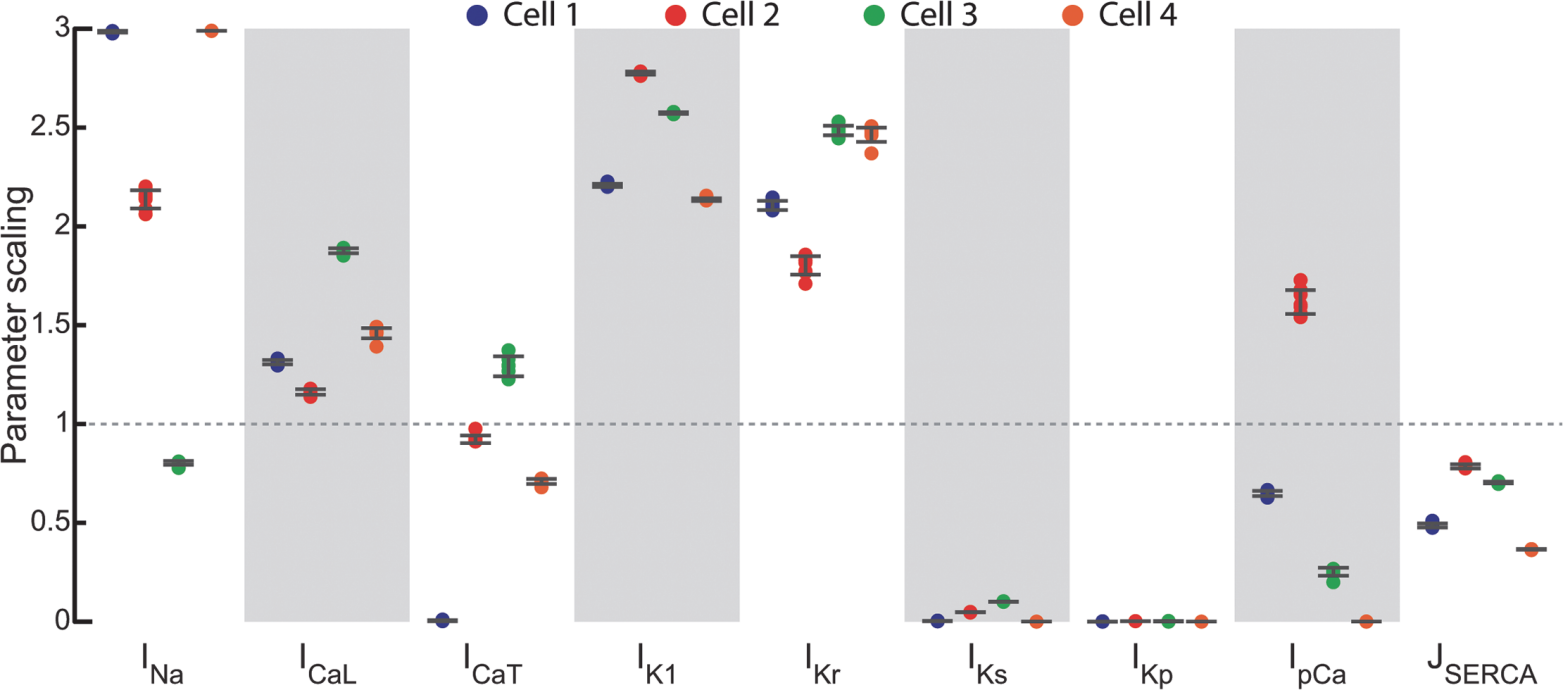
Parameter estimation for individual guinea pig left ventricular myocytes. Estimated parameters from the four isolated myocytes (colored circles; bars give standard deviation) reveal conserved variation (e.g., I_K1_, I_Ks_, I_CaL_) from the original FR model (dashed line) as well as cell-to-cell variability (e.g., I_Na_).

The dissimilarities between the original FR model and the experimental data led to considerable changes in the estimated values for the model parameters for all four cells (Fig 6). Interestingly, these changes were qualitatively similar between all four myocytes for most of the parameters, indicating conserved differences between our experimental data and the FR model. In particular, I_Ks_ and I_Kp_ are scaled down significantly and J_SERCA_ is slightly reduced. In contrast, for all four cells, maximal conductance of I_Kr_ and I_K1_ are increased around 2-fold compared to the FR model, while I_CaL_ is slightly increased. The results for I_Na_ show variation among cells, with a significant increase for three out of four cells and a small decrease for one cell.

In summary, the optimized models show a much closer match to the experimental data as reflected in the individual voltage and current traces as well as in the prediction error. In addition, the optimization identified similar trends in the underlying channel conductance values for different cells from a particular region in the heart. Considered together with the demonstration that the approach accurately identifies model parameters (Figs 2–4), these findings suggest that the approach significantly improves the fidelity of the model for cellular data, relative to the published generic model.

## Discussion

To overcome the limitations inherent to traditional cardiac model construction (most notably manual parameter adjustment and use of averaged, dynamically limited data), we developed a novel approach for parameter estimation that combines an electrophysiology protocol that is rich in dynamic information, short in duration, experimentally feasible, and does not require the use of drugs or special solutions, with a parallel computational parameter tuning algorithm (GA). The protocol was first tested computationally, which showed reliable parameter estimation. We then applied the protocol *in vitro* to guinea pig basal left ventricular myocytes. Compared to the baseline guinea pig FR model, the optimized cell-specific models showed a significantly improved fit to the experimental data. Furthermore, because our model enables validation on data from the same cell for which a model was optimized, we were able to demonstrate that the cell-specific models are markedly better at predicting the response to novel stimulation sequences than was the generic model.

### Complex driving protocols and objectives

In cardiac modeling, a single action potential or biomarkers derived from it such as amplitude and duration, is often used as a minimal objective for model parameterization. Ionic models can be optimized to fit single action potentials using, e.g., global search heuristics [22,23], but because the optimization problem is overdetermined, fits may be improved when adding more data, such as data recorded at multiple pacing rates [19,22]. In fact, relative to a single action potential, more complex driving protocols have the potential to dramatically improve parameterization by creating target objectives that are richer in information. On the other hand, to be experimentally feasible, protocols have to be relatively short in duration due to the inevitable current rundown that occurs in patched myocytes, even when using perforated patch. As a compromise, we utilized a stochastic stimulation protocol because it rapidly samples the rate-dependence of the action potential. In addition, irregular excitation patterns are present in many cardiac arrhythmia; thus models tuned to aperiodic excitation patterns are inherently better suited for modeling irregular arrhythmia. In our simulations, we found that the estimates for I_Kr_ and I_Ks_ were improved the most by the stochastic stimulation objective. During a single action potential, I_Kr_ and I_Ks_ have similar and compensatory effects (S4 Fig), which impedes estimation of their conductances. In contrast, stochastic stimulation more thoroughly explores their kinetics, thereby revealing small differences throughout the protocol, resulting in a more accurate estimation. Although the stochastic stimulation protocol led to at most a modest improvement in the parameter estimation for the remaining parameters, the prediction error was reduced by an order of magnitude, compared to using a single action potential (Fig 2). Thus, significant model improvement is obtained through the use of a dynamically rich objective, as this helps the optimization avoid the false alternatives that can appear to fit well when dynamically sparse data are used for fitting.

In addition to such current-clamp experiments, currents recorded during voltage clamping add additional data to improve fitting and optimization [21,31,32]. While our multi-step voltage clamp protocol alone is very useful for estimating many of the parameter values (Fig 4B), it tends to generate models that fail to predict novel stochastic pacing data well (Fig 4C), which is unsurprising given that it does not train the models according to membrane potential. In our simulations, the addition of the multi-step voltage clamp objective to the stochastic current-clamp stimulation objective enhanced the quality of the parameter estimation compared to using only stochastic stimulation (Fig 4B). This improvement was the result of: (i) some parameters being estimated accurately by the voltage-clamp protocol and (ii) information on two, rather than a single, state variable putting more constraints on the parameter values [36]. In particular, estimates for all nine parameters became centered on their baseline values and the prediction error dropped by another order of magnitude relative to that of stochastic pacing alone. Finally, the iterative optimization approach [31] refined our *in silico* parameter estimation by decreasing the spread of the returned parameter sets, which caused the prediction error to again decrease by an order of magnitude.

### Improvement in model parameterization for intact cardiac myocytes

Generic models have the advantage that direct comparisons can be made among different simulation studies. However, when comparing a generic model such as the out-of-the-box FR model to our experimental data, there are substantial differences, which likely would cause inaccurate predictions if simulating, e.g., effects of pharmacological agents or genetic variations. For one, there are clear distinctions in action potential morphology, e.g., in the plateau phase (Fig 5). This difference in plateau phase most likely explains the method’s downscaling of the I_Kp_ conductance. Our recorded action potentials are also of considerably longer duration, which is consistent with the finding of a much reduced I_Ks_ in the voltage-clamp experiments. The step to -120 mV in the voltage-clamp protocol induced a much larger current in the experiments than in the FR model and I_K1_ conductance was increased accordingly in all four cells. These consistent changes in voltage traces and currents between our cells and the FR model may be due to lab-to-lab variability and to the fact that the FR model is not region-specific.

### Cell-specific models

Despite such consistent changes, the parameterization also points to important cell-to-cell variability, in particular for the I_Na_ conductance, which is increased in three cells and decreased in one. In neuronal modeling, it has become clear that different combinations of conductance parameter sets can give rise to the same activity pattern and that using average values of the conductances may fail to generate that pattern [10,11,37]. The differences in cell-to-cell variation in current densities have been linked to mRNA expression differences or post-translational modifications [38,39]. The extent to which such variation occurs in healthy cardiomyocytes remains to be seen, but some examples of functional coupling among ionic currents in perturbed systems have been described [40–42]. This failure-of-averaging concept may also extend to cardiac tissue: although intrinsic cellular heterogeneity tends to be smoothed out when myocytes are electrically coupled, coupled cells do not necessarily behave like their average. For example, a myocyte with intrinsically shorter action potential duration may promote repolarization in a cell pair [43]. Also, a range of synchronization patterns have been described in coupled pacemaker cells [44]. Thus, there may be important utility to developing cell-specific models.

Indeed, cardiac cell-specific models have a range of potential application areas. First, the models can obviously be used to study cell-to-cell variability [45]. Second, in the clinic, inter-subject variability can lead to response differences among patients to pharmaceutical treatment. A dramatic example of this variation is the response to I_Kr_-block, which can vary from minor changes in the electrocardiogram to ventricular tachyarrhythmias [46]. Understanding and predicting this variability is an important step towards patient-specific treatment. In turn, model optimizations such as those developed here represent an advancement towards patient-specific prediction. Finally, multiple models could be grouped into a heterogeneous population and used to generate more realistic responses than those of a randomly-generated population [47,48].

### Limitations and potential improvements

The developed protocols allow accurate estimation of nine conductance/flux parameters. To characterize a single cell more thoroughly, additional flux parameters could be included (e.g., those describing the sodium/calcium exchanger and the sodium/potassium pump), but as inclusion of more parameters makes the optimization problem harder, this may necessitate tweaking of the methods described here. As detailed below, possible strategies for improvement of the parameter estimations are: 1) improving the stochastic stimulation and voltage-clamp protocols; 2) adding measurements of different state variables during the same protocols (e.g., intracellular calcium or membrane resistance); 3) incorporating altered solutions and/or ion channel blockers to improve isolation of individual currents [49]; 4) including ion channel kinetic parameters in the optimization; or 5) including relative weights for the current and the voltage contributions to the summed error.

Our multi-step voltage-clamp protocol effectively isolates I_Ks_, I_CaL_, and I_K1_. An improved voltage-clamp sequence that isolates the remaining currents could improve estimation of their conductance/flux parameters. We designed the voltage clamp steps based on *a priori* knowledge of the current-voltage (IV) relations in guinea pig ventricular myocytes. As a way to design better protocols, an automated optimization approach may be feasible, i.e., an optimization of the optimization protocol.

Further, differences in structure, channel kinetics and IV-relationships between model and experiment are likely to result in less accurate parameter estimations [31] and may underlie the deviations between fit and experimental data during voltage clamp (Fig 5 and S7–S9 Figs). Adding parameters describing ion channel kinetics to the optimization process would likely improve the fits and predictability, but would almost certainly necessitate longer voltage clamp protocols [6,16]. As channel kinetics are not expected to vary substantially among cells of the same type, a possible strategy is to first parameterize average channel kinetics in a cell population, then apply our method to derive cell specific models.

Additionally, improvement could likely be gained by simultaneously recording calcium fluorescence and adding that to the objective function [36], a strategy with merits illustrated by Fig 1 of Ref. [26]. As expected, local sensitivity analysis on simulated calcium traces demonstrates that they are most sensitive to changes in I_CaL_, I_pCa_, and J_SERCA_ (S5 Fig), which leads to the speculation that the estimation of these parameters could improve. Inclusion of calcium data may also allow determination of I_NaCa_, which depends on and influences both intracellular calcium and transmembrane potential. Incorporation of membrane resistance in the objective function would also be expected to improve the fitting, as shown in recent work by Kaur et al. [25]. A potential caveat in such multi-objective optimization is that simultaneous good fits are not always achievable, necessitating trade-offs between the different objectives. In that case, balancing which objective(s) to prioritize would be application dependent.

Although we allow a generous range for the conductance parameters (0.01–299% of baseline), some parameters did reach the bounds when fitting the experimental data (Fig 6). Increasing the range will likely require running the GA optimization with a larger population size or for more generations, as will including additional parameters. The main computational cost of the GA is that of simulating the individual models. As this process is inherently parallel, it is straightforward to take advantage of parallel computing. Future implementations could decrease run time by utilizing a GPU, on which optimization for neuronal data has been shown to be feasible [32].

Finally, although the four cells tested in this study provide a strong proof-of-concept for the approach, to further develop the method, it could be applied to a larger number of cells.

### Conclusions

In the novel approach developed here, cell-specific cardiac models are developed by coupling complex electrophysiology protocols with genetic algorithm parameter fitting. Neither the electrophysiological data (which are too complex to fit by hand), nor the fitting algorithm, would offer much advantage alone. However, merging the two enables markedly improved models that can more accurately simulate dynamically rich cardiac dynamics than can models developed using traditional approaches. Given the widespread use of ionic cardiomyocyte models in investigating arrhythmogenesis, there is utility in models that are better at reproducing such rich electrophysiological dynamics, which are more representative of the complex dynamics that are often inherent to arrhythmias. In addition to improving model fidelity generally, because this approach can be used to generate a model from a single cardiac myocyte, it may be useful in applications ranging from studying the implications of cell-to-cell variability to the prediction of intersubject differences in response to pharmacological treatment.

## Methods

### Ethics statement

All animal care and handling for this study was performed in strict accordance with the recommendations in the Guide for the Care and Use of Laboratory Animals of the National Institutes of Health. The protocol was approved by the Institutional Animal Care and Use Committee of Weill Cornell Medical College (protocol number: 0701-571A).

### Computational modeling

The cardiac guinea pig model developed by Faber and Rudy ("FR model") [34] was used. Model intracellular and extracellular ionic concentrations were set to the values used in our *in vitro* experiments (see below), after which the model was simulated to a steady state in current clamp mode for 1800 beats at a pacing cycle length of 500 ms. Stimuli were square pulses of 1 ms duration and -40 A/F amplitude.

### Stochastic stimulation protocol

Stochastic stimulation sequences were 5 s in duration. Stimulation times were randomly drawn from a uniform distribution with a range of 100–700 ms. Stimulation times for the optimization sequence were: 216.48, 623.36, 764.64, 1101.12, 1790.16, 2073.10, 2642.28, 3183.10, 3786.07, 3959.02, and 4579.72 ms (Fig 2A). Prediction sequence stimulation times were: 247.40, 705.30, 1020.60, 1347.90, 1462.60, 1705.60, 2038.30, 2546.70, 3150.30, 3706.70, 3939.70, 4077.80, and 4645.50 ms (S6 Fig). After each parameter change during the GA optimization (details below), current clamp was simulated for 9 beats at a static pacing interval of 500 ms to dampen transients.

### Multi-step voltage clamp protocol

The voltage clamp protocol was designed on general, *a priori*, IV relations for the individual channels (e.g., I_K1_ is the predominately active current at a holding potential of -120 mV). The 6000 ms protocol was composed of the following steps: 50 ms at -80 mV, 50 ms at -120 mV, 500 ms at -57 mV, 25 ms at -40 mV, 75 ms at +20 mV, 25 ms at -80 mV, 250 ms at +40 mV, 1900 ms at -30 mV, 750 ms at +40 mV, 1725 ms at -30 mV and 650 ms at -80 mV (Fig 3). The contribution of individual membrane currents to the voltage clamp protocol was evaluated using the FR model and the following equation:

$$Contribution=100\%\cdot \frac{\sum_{t=t_{1}}^{t_{2}}\left|I_{x}(t)\right|}{\sum_{j=1}^{N}\sum_{t=t_{1}}^{t_{2}}\left|I_{j}(t)\right|}$$ (1)

Eq 1 calculates the percentage contribution of the absolute individual current (I_x_) relative to the absolute sum of all N currents for all time points during one of the holding potentials (t_1_ to t_2_). This calculation was done for all model currents at all holding potentials.

During GA optimization, the simulated multi-step voltage clamp is preceded by 5 s holding at -80 mV to allow the model to settle after parameter changes.

### Genetic algorithm optimization

Multiple global search heuristics have been applied to electrophysiology models, including gradient-based descent [13,19–21], simulated annealing [50], particle swarms [18,24], and genetic algorithms [22,23,25]. We chose a genetic algorithm as it is effective for a range of the number of parameters [50], is computationally simple and readily parallelizable, and has been shown to be successful at optimizing sophisticated ionic models to experimental data [22,23,25].

The GA used in this study was originally developed by Sastry [33] and was used with the settings for selection, crossover, mutation, and elitism strategy as in Bot et al. [23]. Compared to the study of Bot et al., we increased the parameter search range to 0.01–299% of the baseline model values. This larger range, in combination with the increased number of parameters and the diminished requirements for computation speed relative to the Bot et al. study, caused us to enlarge the population size to 500 and raise the number of generations to 100, based on test runs showing consistent convergence when using these values. Because the GA is inherently stochastic, it was run 10 times per optimization problem. In addition, an iterative approach was implemented, based on the study of Hobbs and Hooper [31]. For each parameter, the iterative approach uses the span of the 10 best individuals from the first 10 GA runs as the search boundaries for a second set of GA runs. With these new search ranges, the GA was again run 10 times with a population of 500 individuals for 100 generations.

We used mean squared differences for the objective functions (errors) that the GA works to minimize:

$$E_{1}=\sum_{t=t_{IC,start}}^{t_{IC,end}}{\left(V_{target}\left(t\right)-V_{individual}\left(t\right)\right)}^{2}$$ (2)

$$E_{2}=\sum_{t=t_{IC,start}}^{t_{IC,end}}{\left(V_{target}\left(t\right)-V_{individual}\left(t\right)\right)}^{2}+\sum_{t=t_{VC,start}}^{t_{VC,end}}{\left(I_{target}\left(t\right)-I_{individual}\left(t\right)\right)}^{2}$$ (3)

where E_1_ is the objective function when only current clamp data (i.e., stochastic stimulation or a single action potential) was fit, and E_2_ is the objective function for the combined stochastic stimulation and multi-step voltage clamp protocol. In both equations, V_target_ is the membrane potential during current clamp of the target (i.e., either the simulated nominal model or the experimental data), and V_individual_ is the membrane potential of a simulated individual. I_target_ and I_individual_ are the current responses during voltage clamp of the target and a simulated individual, respectively. Errors are summed over the entire duration of the protocols.

Although of different units, the voltage clamp and the current clamp components to E_2_ were simply summed into a single objective (Eq 3) as we expect them to be minimal for the same range of parameters, rather than being competitive as in typical multi-objective optimization. E_2_ is therefore unitless.

The estimated model parameters are the maximal conductances of I_Na_, I_CaL_, I_CaT_, I_K1_, I_Kr_, I_Ks_, I_Kp_, and I_pCa_, and the maximal flux of J_SERCA_.

Optimizations were run on a 3.2Ghz Intel Xeon W3670 6-core, 6GB memory, machine and took approximately 8 hours per run for the iterative approach and then combined stochastic pacing and voltage clamp protocol.

### Statistics

Two sample t-tests were performed with a significance level of 0.05. Numbers and error bars indicate average ± standard deviation.

### Electrophysiology experiments

Guinea pigs (n = 4) were anesthetized using an intraperitoneal injection with Euthasol (Virbac Corporation, Fort Worth, TX), 550 mg/kg. Excised hearts were then Langendorff retrograde perfused, and myocytes were isolated from the base (top 1/3) of the left ventricle through enzymatic digestion. Myocytes were stored in Dulbecco’s Modified Eagle Medium (DMEM) with 5% fetal bovine serum (FBS).

Amphotericin-B (Sigma-Aldrich Corp., St. Louis, MO; 480 μg per 1 ml pipette solution) perforated patch clamp technique was used to record cellular action potentials. Bath solution contained (in mmol/l) 139.4 NaCl, 5.4 KCl, 1.0 MgSO_4_, 10.0 Hepes, 10.0 dextrose, 2.0 CaCl_2_, pH 7.35 with NaOH, osmolality 310 ± 3 mmol/kg. Intracellular solution contained (in mmol/l unless otherwise noted) 125 KCl, 10 NaCl, 5.5 dextrose, 0.5 MgCl_2_, 11 KOH, 10 Hepes, 10 μmol/l CaCl_2_, pH 7.1 with HCl, osmolality 295 ± 3 mmol/kg. Recordings were performed at 35°C.

Patch-clamp measurements were recorded using an Axopatch 200A amplifier (Molecular Devices, Sunnyvale, CA). The Real-Time eXperiment Interface [RTXI; rtxi.org; [51,52]] software platform was used to control the amplifier and record data. Cells were initially paced in current clamp mode at a BCL of 500 ms to steady state (500–1000) beats using suprathreshold square pulses of 1 ms duration. Next, the optimization and prediction stochastic stimulation sequences were applied. Amplifier mode was then switched to voltage clamp and series resistance measured (4–8 MΩ) and compensated for (70–90%). The multi-step voltage clamp protocol was then applied in triplet. Holding potentials were corrected for a liquid junction potential of -3 mV. The magnitude of the Donnan equilibrium was estimated to 0 mV using the IV-curve of I_Na_ and therefore not corrected for.

### Post-processing of experimental data

To remove stimulus artifacts from current-clamp traces, data from a 1.3 ms window following the start of each stimulus were excluded from the optimization. In addition, voltage-clamp data from a 1.2 ms window following each potential change were excluded from the GA optimization because of the capacitance transient.

From the set of three voltage-clamp trials, the current response trace with the shortest time to peak I_Na_ (step to -40 mV at 600 ms) was selected for each cell.

## Supporting Information

S1 Text Supplemental results. Methods and results of sensitivity and correlation analyses.
(PDF)

S1 Fig Local sensitivity analysis of FR model during stochastic pacing, voltage clamp, and combined protocol. Parameters were scaled to 80, 90, 95, 105, 110 and 120% of their published values and the sum of squared errors was calculated and visualized here as the sensitivity. For each parameter, the effect of the scaling is given from small to large parameter scaling, i.e., from 80–120%. See S1 Text for details.
(PDF)

S2 Fig Multi-parameter sensitivity analysis of FR model during single action potential, stochastic pacing, and combined protocol. Multi-parameter sensitivity analysis was carried out as detailed in S1 Text. Blue symbols indicate parameters with a statistically significant effect on the model output while the red symbols indicate the parameters with a non-significant effect on the model output (I_CaT_, I_Kp,_ and I_pCa_). The dashed line indicates the largest sensitivity of the non-significant parameters and is visualized as a threshold value for sensitivity.
(PDF)

S3 Fig Progression analysis for single action potential and stochastic pacing. The figures give the squared coefficient of variation (standard deviation normalized to the mean) for each conductance/flux parameter during the optimization process, averaged for the 10 GA runs. Slow convergence indicates less sensitivity. See S1 Text for details.
(PDF)

S4 Fig Linear correlation analysis of FR model during single action potential, stochastic pacing and combined protocol. Colors represent the value of the correlation between two parameters. Symbols indicate statistical significance. See S1 Text for details.
(PDF)

S5 Fig Local sensitivity analysis of FR model calcium dynamics during stochastic pacing and voltage clamp protocol. Parameters were scaled to 80, 90, 95, 105, 110 and 120% of the published value and the sum of squared errors (using intracellular calcium concentration rather than transmembrane potential or total current in Eqs 2 and 3) was calculated and visualized here as the sensitivity. For each parameter, the effect of the scaling is given from small to large parameter scaling, i.e., from 80–120%. The calcium signal is most sensitive to parameters that are directly calcium-related (I_CaL_, J_SERCA_, and I_pCa_).
(PDF)

S6 Fig Stochastic pacing prediction. A) Prediction sequence used to calculate prediction error. Best individual from stochastic pacing GA optimization runs (blue, dashed) and FR model (black) show close correspondence. B) Prediction error calculation for the best individual from 10 GA optimization runs using a single action potential (green) or stochastic pacing (blue). FR model simulation (objective) is given in black. The individual from the stochastic pacing runs matches the FR objective more closely.
(PDF)

S7 Fig Experimental data fit, cell 1. Stochastic pacing and voltage clamp fits of the experimental data of cell 1. The figure shows the best individual from 10 GA runs using the iterative approach (blue), the original FR model (black) and the experimental data (red). The GA fit shows a closer match with the experimental data than the FR model. Stimulus artifacts and capacitative currents were removed (as in Fig 5), but data sets were plotted as continuous traces to ease visualization.
(PNG)

S8 Fig Experimental data fit, cell 3. Stochastic pacing and voltage clamp fits (blue) of the experimental data of cell 3 (red) compared to the original FR model (black).
(PNG)

S9 Fig Experimental data fit, cell 4. Stochastic pacing and voltage clamp fits (blue) of the experimental data of cell 4 (red) compared to the original FR model (black).
(PNG)

## References

[1] Hodgkin AL, Huxley AF (1952) A quantitative description of membrane current and its application to conduction and excitation in nerve. J Physiol 117: 500–544. 12991237 10.1113/jphysiol.1952.sp004764 PMC1392413

[2] Bueno-Orovio A, Sánchez C, Pueyo E, Rodriguez B (2014) Na/K pump regulation of cardiac repolarization: insights from a systems biology approach. Pflugers Arch 466: 183–193. 10.1007/s00424-013-1293-1 23674099

[3] Noble D, Garny A, Noble PJ (2012) How the Hodgkin-Huxley equations inspired the cardiac Physiome Project. J Physiol 590: 2613–2628. 10.1113/jphysiol.2011.224238 22473779 PMC3424720

[4] Rudy Y (2012) From Genes and Molecules to Organs and Organisms In: Edward H. Egelman, editor: Comprehensive Biophysics, Vol 9, Simulation and Modeling. Oxford: Academic Press, pp. 268–327.

[5] Krogh-Madsen T, Christini DJ (2012) Nonlinear Dynamics in Cardiology. Annu Rev Biomed Eng 14: 179–203. 10.1146/annurev-bioeng-071811-150106 22524390 PMC3733460

[6] Zhou Q, Zygmunt AC, Cordeiro JM, Siso-Nadal F, Miller RE, Buzzard GT, et al (2009) Identification of I_Kr_ kinetics and drug binding in native myocytes. Ann Biomed Eng 37: 1294–1309. 10.1007/s10439-009-9690-5 19353268 PMC2690829

[7] Fink M, Niederer SA, Cherry EM, Fenton FH, Koivumäki JT, Seemann G, et al (2011) Cardiac cell modelling: Observations from the heart of the cardiac physiome project. Prog Biophys Mol Biol 104: 2–21. 10.1016/j.pbiomolbio.2010.03.002 20303361

[8] Niederer SA, Fink M, Noble D, Smith NP (2009) A meta-analysis of cardiac electrophysiology computational models. Exp Physiol 94: 486–495. 10.1113/expphysiol.2008.044610 19139063

[9] Krogh-Madsen T, Schaffer P, Skriver AD, Taylor LK, Pelzmann B, Koidl B, et al (2005) An ionic model for rhythmic activity in small clusters of embryonic chick ventricular cells. Am J Physiol Heart Circ Physiol 289: H398–H413. 15708964 10.1152/ajpheart.00683.2004

[10] Golowasch J, Goldman MS, Abbott LF, Marder E (2002) Failure of averaging in the construction of a conductance-based neuron model. J Neurophysiol 87: 1129–1131. 11826077 10.1152/jn.00412.2001

[11] Marder E (2011) Variability, compensation, and modulation in neurons and circuits. Proc Natl Acad Sci USA 108 Suppl 3: 15542–15548. 10.1073/pnas.1010674108 21383190 PMC3176600

[12] Gemmell P, Burrage K, Rodriguez B, Quinn TA (2014) Population of computational rabbit-specific ventricular action potential models for investigating sources of variability incellular repolarisation. PLoS ONE 9: e90112 10.1371/journal.pone.0090112 24587229 PMC3938586

[13] Bueno-Orovio A, Cherry EM, Fenton FH (2008) Minimal model for human ventricular action potentials in tissue. J Theor Biol 253: 544–560. 10.1016/j.jtbi.2008.03.029 18495166

[14] Wilhelms MM, Hettmann HH, Maleckar MMM, Koivumäki JTJ, Dössel OO, Seemann G (2012) Benchmarking electrophysiological models of human atrial myocytes. Front Physio 3: 487–487. 10.3389/fphys.2012.00487 PMC3539682 23316167

[15] Vecchietti S, Rivolta I, Severi S, Napolitano C, Priori SG, Cavalcanti S (2006) Computer simulation of wild-type and mutant human cardiac Na^+^ current. Med Bio Eng Comput 44: 35–44. 16929919 10.1007/s11517-005-0017-x

[16] Fink M, Noble D (2009) Markov models for ion channels: Versatility versus identifiability and speed. Philos Transact A Math Phys Eng Sci 367: 2161–2179. 10.1098/rsta.2008.0301 19414451

[17] Moreno JD, Zhu ZI, Yang P-C, Bankston JR, Jeng M-T, Kang C, et al (2011) A computational model to predict the effects of class I anti-arrhythmic drugs on ventricular rhythms. Science Trans Med 3: 1–9. 10.1126/scitranslmed.3002588 PMC3328405 21885405

[18] Weber FM, Lurz S, Keller D, Weiss DL, Seemann G, Lorenz C, et al (2008) Adaptation of a minimal four-state cell model for reproducing atrial excitation properties. Comput Cardiol 35: 61–64.

[19] Guo TT, AA Abed Al A, Lovell NHN, Dokos SS (2010) A generic ionic model of cardiac action potentials. IEEE Eng Med Biol Soc 2010: 1465–1468. 10.1109/IEMBS.2010.5626853 21096358

[20] Guo T, Abed Al A, Lovell NH, Dokos S (2013) Optimisation of a generic ionic model of cardiac myocyte electrical activity. Comput Math Methods Med 2013: 706195—1–20. 10.1155/2013/706195 PMC3659483 23710254

[21] Dokos S, Lovell NH (2004) Parameter estimation in cardiac ionic models. Prog Biophys Mol Biol 85: 407–431. 15142755 10.1016/j.pbiomolbio.2004.02.002

[22] Syed Z, Vigmond E, Nattel S, Leon LJ (2005) Atrial cell action potential parameter fitting using genetic algorithms. Med Bio Eng Comput 43: 561–571. 16411628 10.1007/BF02351029

[23] Bot CT, Kherlopian AR, Ortega FA, Christini DJ, Krogh-Madsen T (2012) Rapid genetic algorithm optimization of a mouse computational model: Benefits for anthropomorphization of neonatal mouse cardiomyocytes. Front Physio 3: 1–14. 10.3389/fphys.2012.00421 PMC3488799 23133423

[24] Chen F, Chu A, Yang X, Lei Y, Chu J (2012) Identification of the parameters of the Beeler-Reuter ionic equation with a partially perturbed particle swarm optimization. IEEE Trans Biomed Eng 59: 3412–3421. 10.1109/TBME.2012.2216265 22955867

[25] Kaur J, Nygren A, Vigmond EJ (2014) Fitting membrane resistance along with action potential shape in cardiac myocytes improves convergence: Application of a multi-objective parallel genetic algorithm. PLoS ONE 9: e107984 10.1371/journal.pone.0107984 25250956 PMC4176019

[26] Sarkar AX, Sobie EA (2010) Regression analysis for constraining free parameters in electrophysiological models of cardiac cells. PLoS Comput Biol 6: e1000914 10.1371/journal.pcbi.1000914 20824123 PMC2932676

[27] Milescu LS, Yamanishi T, Ptak K, Mogri MZ, Smith JC (2008) Real-time kinetic modeling of voltage-gated ion channels using dynamic clamp. Biophys J 95: 66–87. 10.1529/biophysj.107.118190 18375511 PMC2426646

[28] Gurkiewicz M, Korngreen A (2007) A numerical approach to ion channel modelling using whole-cell voltage-clamp recordings and a genetic algorithm. PLoS Comput Biol 3: e169 17784781 10.1371/journal.pcbi.0030169 PMC1963494

[29] Csercsik D, Hangos KM, Szederkényi G (2012) Identifiability analysis and parameter estimation of a single Hodgkin–Huxley type voltage dependent ion channel under voltage step measurement conditions. Neurocomputing 77: 178–188.

[30] Achard P, De Schutter E (2006) Complex parameter landscape for a complex neuron model. PLoS Comput Biol 2: 0794–0804. 10.1371/journal.pcbi.0020094 PMC1513272 16848639

[31] Hobbs KH, Hooper SL (2008) Using complicated, wide dynamic range driving to develop models of single neurons in single recording sessions. J Neurophysiol 99: 1871–1883. 10.1152/jn.00032.2008 18256169

[32] Tomaiuolo M, Bertram R, Leng G, Tabak J (2012) Models of electrical activity: Calibration and prediction testing on the same cell. Biophys J 103: 2021–2032. 10.1016/j.bpj.2012.09.034 23199930 PMC3491713

[33] Sastry K (2007) Single and Multiobjective Genetic Algorithm Toolbox in C. IlliGAL Report 2007016: 1–14. https://hec.unil.ch/docs/files/6/24/2007016.pdf [Accessed April 16, 2014].

[34] Faber GM, Rudy Y (2000) Action potential and contractility changes in [Na^+^]_i_ overloaded cardiac myocytes: A simulation study. Biophys J 78: 2392–2404. 10777735 10.1016/S0006-3495(00)76783-X PMC1300828

[35] Kalb SS, Dobrovolny HM, Tolkacheva EG, Idriss SF, Krassowska W, Gauthier DJ (2004) The restitution portrait: A new method for investigating rate-dependent restitution. J Cardiovasc Electrophysiol 15: 698–709. 15175067 10.1046/j.1540-8167.2004.03550.x

[36] Raue A, Becker V, Klingmüller U, Timmer J (2010) Identifiability and observability analysis for experimental design in nonlinear dynamical models. Chaos 20: 045105 10.1063/1.3528102 21198117

[37] Weiss JN, Karma A, MacLellan WR, Deng M, Rau CD, Rees CM, et al (2012) “Good enough solutions” and the genetics of complex diseases. Circ Res 111: 493–504. 10.1161/CIRCRESAHA.112.269084 22859671 PMC3428228

[38] Schulz DJ, Goaillard J-M, Marder E (2006) Variable channel expression in identified single and electrically coupled neurons in different animals. Nat Neurosci 9: 356–362. 16444270 10.1038/nn1639

[39] Veys K, Labro AJ, De Schutter E, Snyders DJ (2012) Quantitative single-cell ion-channel gene expression profiling through an improved qRT-PCR technique combined with whole cell patch clamp. J Neurosci Meth 209: 227–234. 10.1016/j.jneumeth.2012.06.008 22728251

[40] Milstein ML, Musa H, Balbuena DP, Anumonwo JM, Auerbach DS, Furspan PB, et al (2012) Dynamic reciprocity of sodium and potassium channel expression in a macromolecular complex controls cardiac excitability and arrhythmia. Proc Natl Acad Sci USA 109: E2134–E2143. 10.1073/pnas.1109370109 22509027 PMC3412015

[41] Deschênes I, Armoundas AA, Jones SP, Tomaselli GF (2008) Post-transcriptional gene silencing of KChIP2 and Na_v_ß1 in neonatal rat cardiac myocytes reveals a functional association between Na and I_to_ currents. J Mol Cell Cardiol 45: 336–346. 10.1016/j.yjmcc.2008.05.001 18565539 PMC2580777

[42] Xiao L, Xiao J, Luo X, Lin H, Wang Z, Nattel S (2008) Feedback remodeling of cardiac potassium current expression: A novel potential mechanism for control of repolarization reserve. Circulation 118: 983–992. 10.1161/CIRCULATIONAHA.107.758672 18711016

[43] Zaniboni M, Pollard AE, Yang L, Spitzer KW (2000) Beat-to-beat repolarization variability in ventricular myocytes and its suppression by electrical coupling. Am J Physiol Heart Circ Physiol 278: H677–H687. 10710334 10.1152/ajpheart.2000.278.3.H677

[44] DeHaan RL, Hirakow R (1972) Synchronization of pulsation rates in isolated cardiac myocytes. Exp Cell Res 70: 214–220. 5008399 10.1016/0014-4827(72)90199-1

[45] Sarkar AX, Christini DJ, Sobie EA (2012) Exploiting mathematical models to illuminate electrophysiological variability between individuals. J Physiol 590: 2555–2567. 10.1113/jphysiol.2011.223313 22495591 PMC3424714

[46] Kannankeril P, Roden DM, Darbar D (2010) Drug-Induced Long QT Syndrome. Pharmacol Rev 62: 760–781. 10.1124/pr.110.003723 21079043 PMC2993258

[47] Britton OJ, Bueno-Orovio A, Van Ammel K, Lu HR, Towart R, Gallacher DJ, et al (2013) Experimentally calibrated population of models predicts and explains intersubject variability in cardiac cellular electrophysiology. Proc Natl Acad Sci USA 110: E2098–E2105. 10.1073/pnas.1304382110 23690584 PMC3677477

[48] Vik JO, Gjuvsland AB, Li L, Tøndel K, Niederer S, Smith NP, et al (2011) Genotype-phenotype map characteristics of an in silico heart cell. Front Physiol 2: 106 10.3389/fphys.2011.00106 22232604 PMC3246639

[49] Banyasz T, Horvath B, Jian Z, Izu LT, Chen-Izu Y (2011) Sequential dissection of multiple ionic currents in single cardiac myocytes under action potential-clamp. J Mol Cell Cardiol 50: 578–581. 10.1016/j.yjmcc.2010.12.020 21215755 PMC3047417

[50] Vanier MC, Bower JM (1999) A comparative survey of automated parameter-search methods for compartmental neural models. J Comput Neurosci 7: 149–171. 10515252 10.1023/a:1008972005316

[51] Lin RJ, Bettencourt J, White JW, Christini DJ, Butera RJ (2010) Real-time experiment interface for biological control applications. IEEE Eng Med Biol Soc 2010: 4160–4163. 10.1109/IEMBS.2010.5627397 21096883 PMC3647344

[52] Ortega FA, Butera RJ, Christini DJ, White JA, Dorval AD (2014) Dynamic Clamp in Cardiac and Neuronal Systems Using RTXI Methods in Molecular Biology. Methods in Molecular Biology. New York, NY: Springer New York, Vol. 1183 pp. 327–354. 10.1007/978-1-4939-1096-0_21 25023319 PMC4880480

