# Supplementary material for: Cell-Specific Cardiac Electrophysiology Models
Source: PLoS Comput Biol. 2015 Apr 30;11(4):e1004242. doi: 10.1371/journal.pcbi.1004242 (PMC4415772; doi:10.1371/journal.pcbi.1004242)
Supplement: S1 Text — Methods and results of sensitivity and correlation analyses. (PDF) [file pcbi.1004242.s001.pdf]

Supporting Information (S1 Text) for

Cell-Specific Cardiac Electrophysiology Models

Willemijn Groenendaal, Francis A. Ortega, Armen R. Kherlopian,
Andrew C. Zygmunt, Trine Krogh-Madsen, and David J. Christini

---

Sensitivity and correlation analysis

Methods

To evaluate the effect of parameter perturbations on membrane potential and membrane current, a local sensitivity analysis was performed. Parameters were individually varied from -20, -10, -5, +5, +10, to +20% of their baseline values and the model was simulated with the changed parameter according to the stochastic pacing, the voltage clamp, or the combined protocol. For each change in parameter, a change in error was calculated from Eqns. 2-3 in the main manuscript or the second term in Eqn. 3 (stochastic pacing, combined protocol, or voltage clamp, respectively). A large change indicates a sensitive parameter.

To assess the parameter sensitivities more globally in parameter space, a multi-parameter sensitivity analysis was performed (1). We used the first 500 randomly generated GA individuals from all 10 GA runs per optimization problem. The individuals were sorted between 'good' and 'bad' individuals, defined as the 10% of the individuals with the lowest error value and the remaining 90% of the individuals, respectively. The parameter distributions between the 'good' and the 'bad' groups were compared using a two-sided Kolmogorov-Smirnov test with a significance level of 0.05. The value of the test statistic is a measure of parameter sensitivity.

Another way to estimate parameter sensitivity is by the method described by Marseguerra et al. (2). This method uses the information within a single GA run to probe how quickly a parameter converges to its final value, taking the rate of convergence as a measure of sensitivity. To do so, a library of the best 100 individuals is made and updated per generation. The variance in parameter value within those 100 best individuals is calculated per generation, with a higher variance indicating slower convergence and a less sensitive parameter.

Because the ionic fluxes controlled by the model parameters are not independent, we performed a parameter correlation analysis. After 10 GA runs for a single optimization problem, correlations between the estimated parameters were studied using Pearson's linear correlation coefficient and a significance level of 0.05.

Results

Sensitivity and correlation analyses were performed to obtain further understanding of the

optimization results. The local sensitivity analysis shows that changes in all parameter values have some effects on the predicted membrane potential during stochastic pacing (S1 Figure). However, the model is relatively insensitive to the I_{CaT} and I_{Kp} conductances and very sensitive the conductances of I_{CaL}, I_{Ks}, and I_{Kr}. In line with this, the results of the multi-parameter sensitivity analysis show that I_{CaT}, I_{Kp}, and also I_{pCa} have non-significant effects on the output, while changes in I_{CaL} and I_{Ks} show the largest effect on the membrane potential (S2 Figure). Similar results are seen for the progression analysis (S3 Figure) with the addition of slow and/or less conversion (corresponding to little sensitivity) for I_{Kr}. These analyses thus demonstrate a relative lack of sensitivity for those parameters that were less well identified in the optimization process (Fig. 2C).

For parameter estimation, it is not only important that the model output is sensitive to a change in a parameter value, but also that the change in output is unique. If changes in two different parameter values have a similar effect on the model output, compensation and incorrect parameter estimation could occur. Hence, during a single action potential, the presence of a positive correlation between the I_{CaL} and I_{Ks} conductances (S4 Figure) could explain why they are the two most sensitive parameters, but are estimated incorrectly. During stochastic pacing this correlation becomes insignificant, as does the negative correlation between I_{Kr} and I_{Ks}, which may explain why these parameters are better estimated when optimizing to the stochastic pacing objective.

The voltage clamp protocol, and hence also the combined protocol, is very sensitive to I_{Ks} (S1-2 Figures). The progression analysis also showed a dominant effect of I_{Ks} on the combined protocol (S3 Figure), which matches the very tight error bars of the estimation of I_{Ks} (Fig. 4A). For the combined protocol, there is still little sensitivity to I_{Kp}, I_{CaT}, and I_{pCa} (S1-3 Figures). Consistent with these findings, the GA estimation results of these parameters show a larger spread around 1.

Interestingly, although J_{SERCA} is not a sarcolemmal current and therefore does not directly contribute to the output variables of transmembrane voltage and total transmembrane current, the voltage is still sensitive to J_{SERCA} and its flux parameter is well estimated. This emphasizes the fact that the cell is a complex system with inherent interdependencies between variables: in this example J_{SERCA} influences intracellular calcium concentration, which in turn regulates I_{CaL}, I_{CaT}, and I_{pCa} and thus transmembrane potential.

References

1. Zi Z (2011) Sensitivity analysis approaches applied to systems biology models. *IET Systems Biology* 5:336–346.
2. Marseguerra M, Zio E, Podofillini L (2003) Model parameters estimation and sensitivity by genetic algorithms. *Annals of Nuclear Energy* 30:1437–1456.
