# Supplementary figures and images for: Cell-Specific Cardiac Electrophysiology Models
Source: PLoS Comput Biol. 2015 Apr 30;11(4):e1004242. doi: 10.1371/journal.pcbi.1004242 (PMC4415772; doi:10.1371/journal.pcbi.1004242)

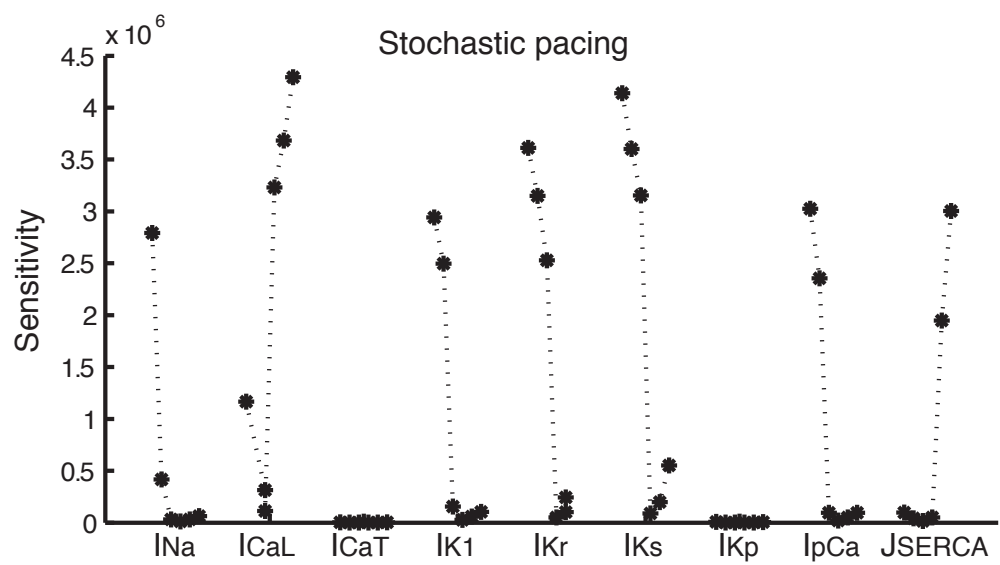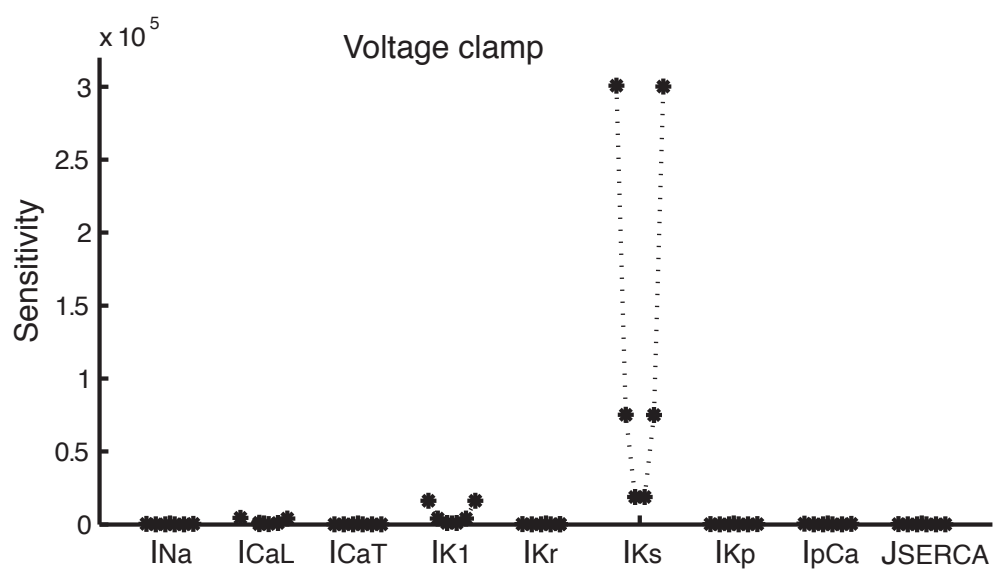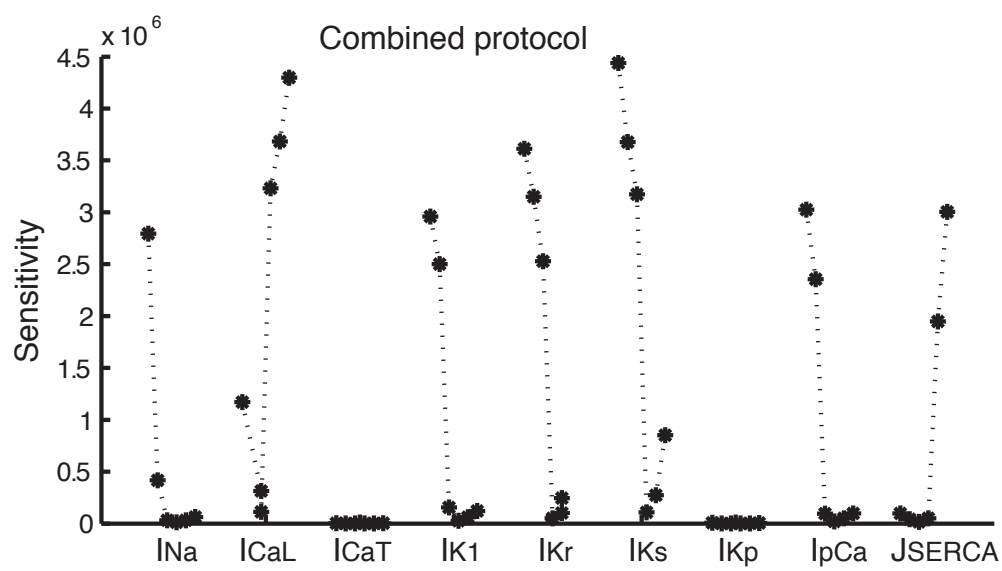

Supplement: S1 Fig — Parameters were scaled to 80, 90, 95, 105, 110 and 120% of their published values and the sum of squared errors was calculated and visualized here as the sensitivity. For each parameter, the effect of the scaling is given from small to large parameter scaling, i.e., from 80–120%. See S1 Text for details. (PDF) [file pcbi.1004242.s002.pdf]

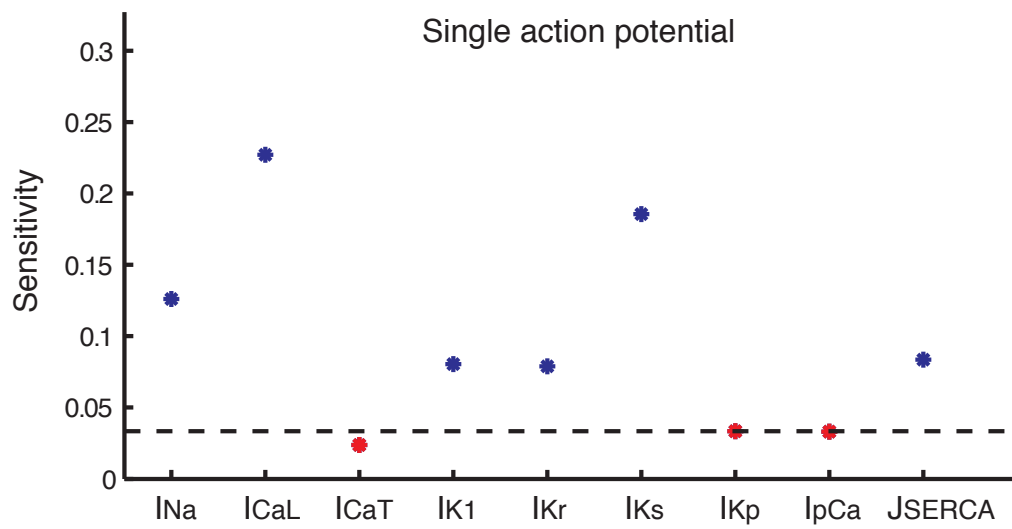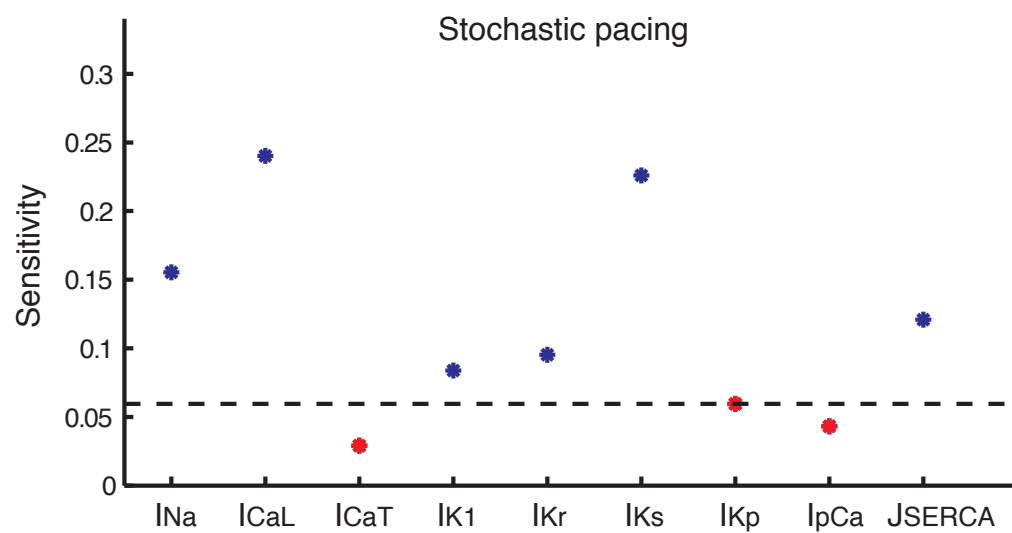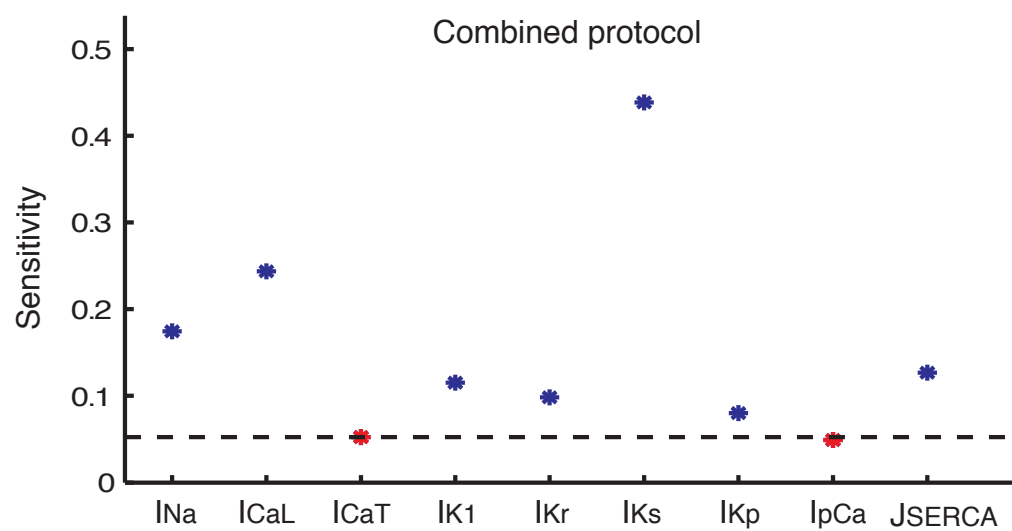

Supplement: S2 Fig — Multi-parameter sensitivity analysis was carried out as detailed in S1 Text. Blue symbols indicate parameters with a statistically significant effect on the model output while the red symbols indicate the parameters with a non-significant effect on the model output (ICaT, IKp, and IpCa). The dashed line indicates the largest sensitivity of the non-significant parameters and is visualized as a threshold value for sensitivity. (PDF) [file pcbi.1004242.s003.pdf]

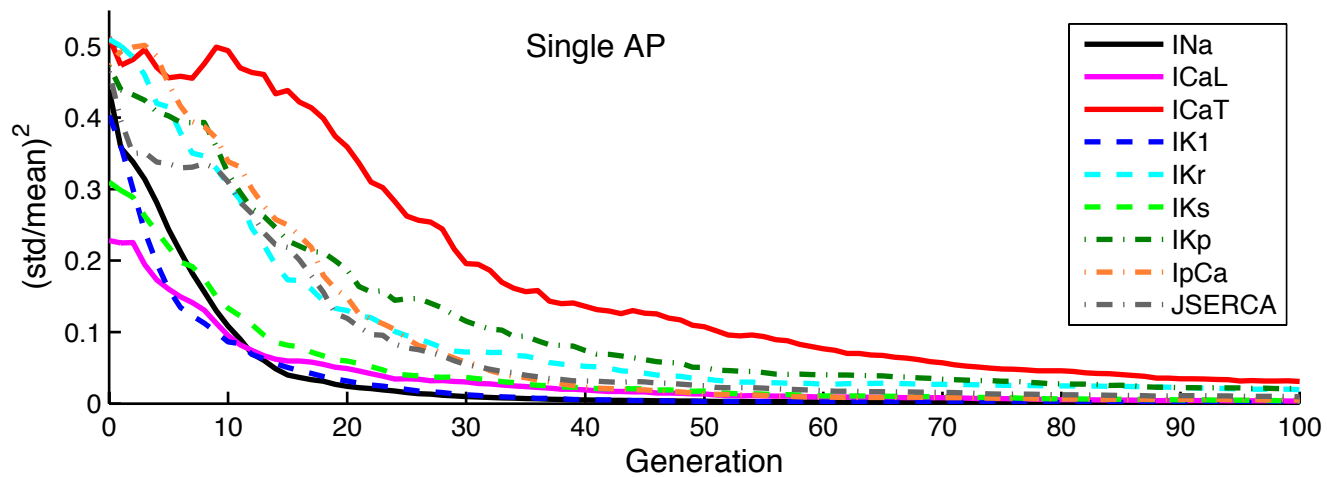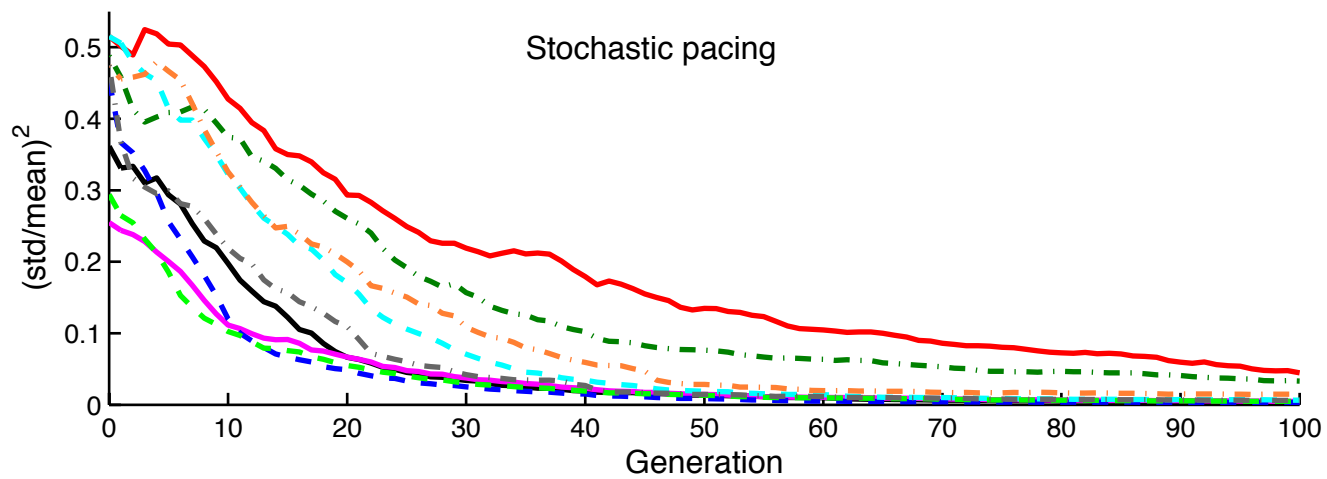

Supplement: S3 Fig — The figures give the squared coefficient of variation (standard deviation normalized to the mean) for each conductance/flux parameter during the optimization process, averaged for the 10 GA runs. Slow convergence indicates less sensitivity. See S1 Text for details. (PDF) [file pcbi.1004242.s004.pdf]

Single AP

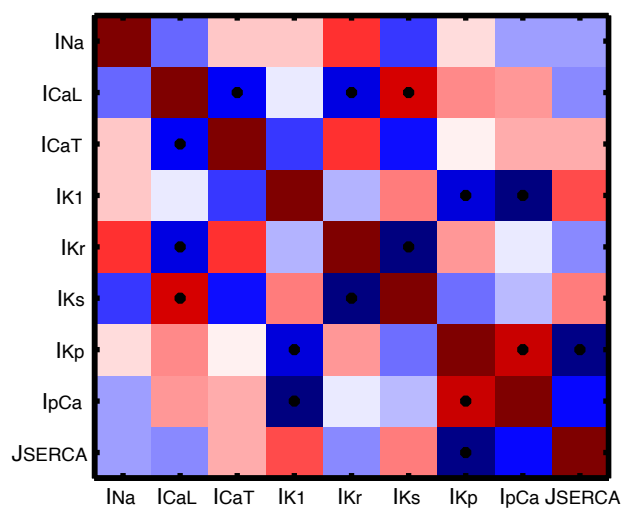

Stochastic pacing

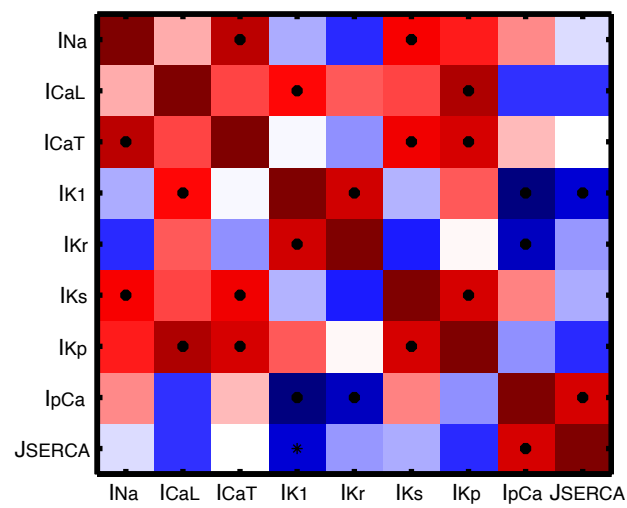

Combined protocol

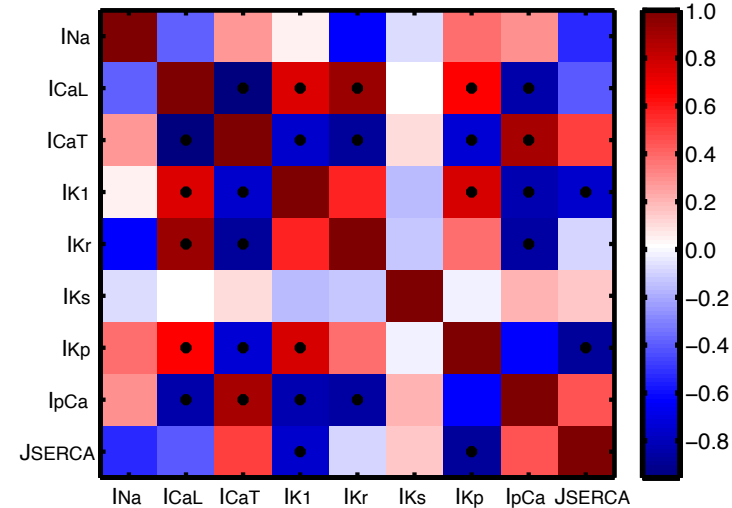

Supplement: S4 Fig — Colors represent the value of the correlation between two parameters. Symbols indicate statistical significance. See S1 Text for details. (PDF) [file pcbi.1004242.s005.pdf]

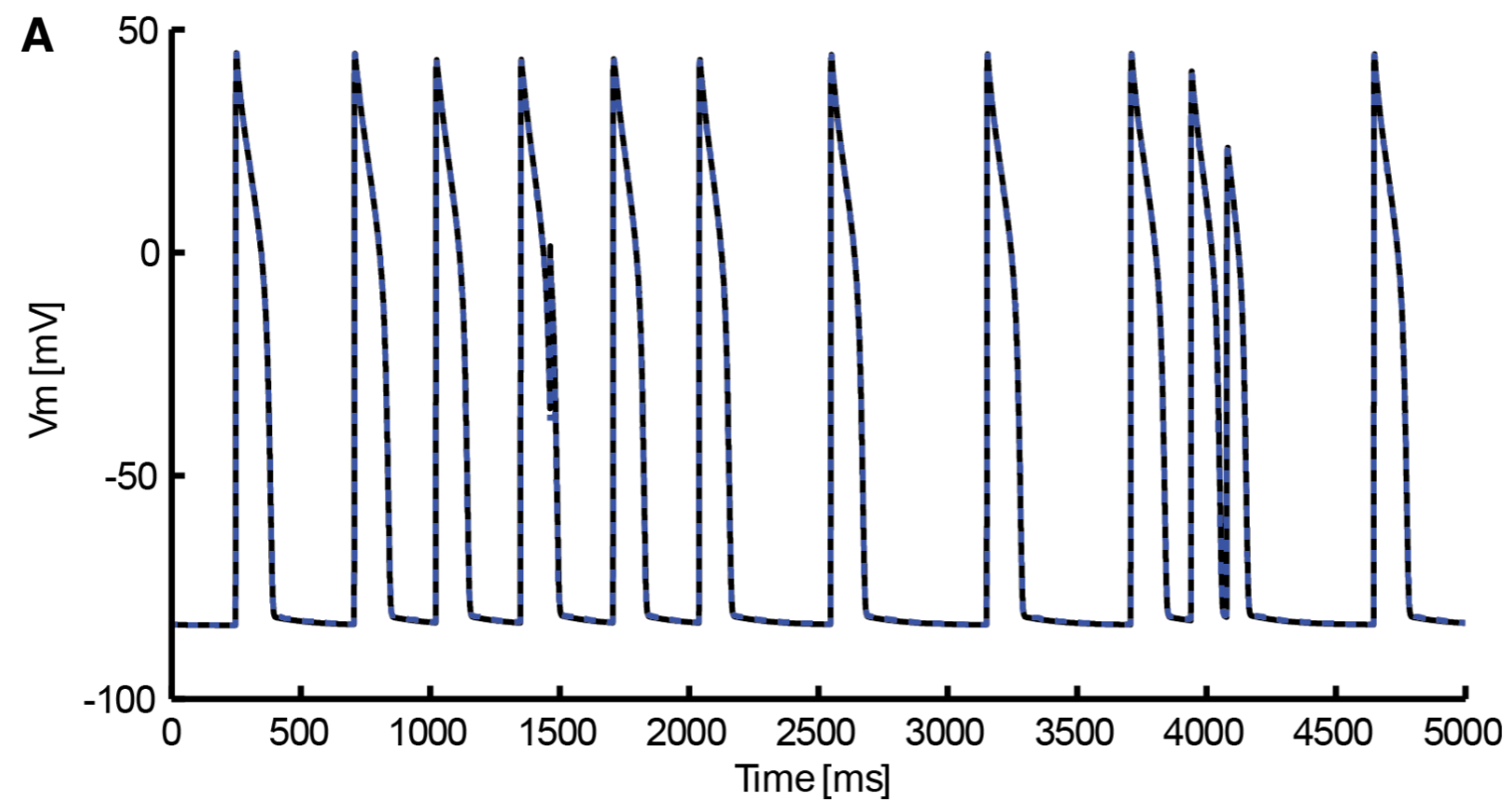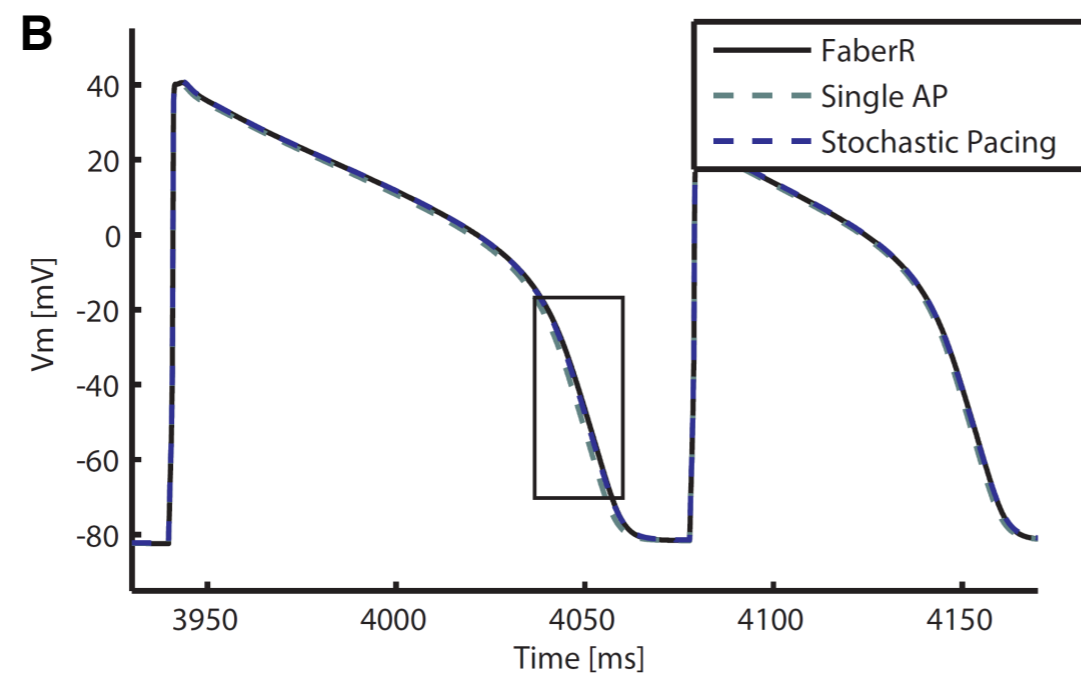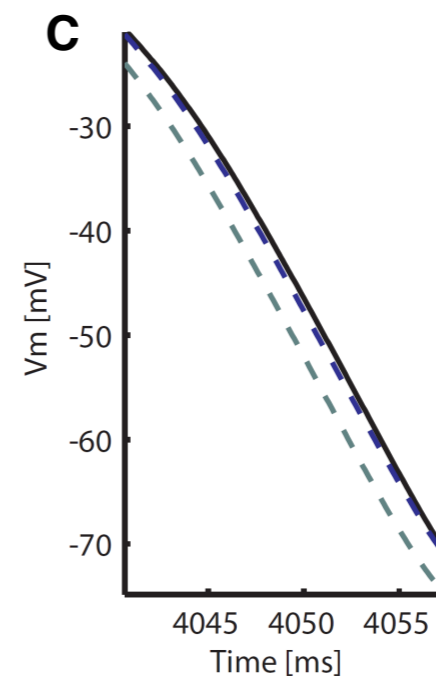

Supplement: S6 Fig — A) Prediction sequence used to calculate prediction error. Best individual from stochastic pacing GA optimization runs (blue, dashed) and FR model (black) show close correspondence. B) Prediction error calculation for the best individual from 10 GA optimization runs using a single action potential (green) or stochastic pacing (blue). FR model simulation (objective) is given in black. The individual from the stochastic pacing runs matches the FR objective more closely. (PDF) [file pcbi.1004242.s007.pdf]

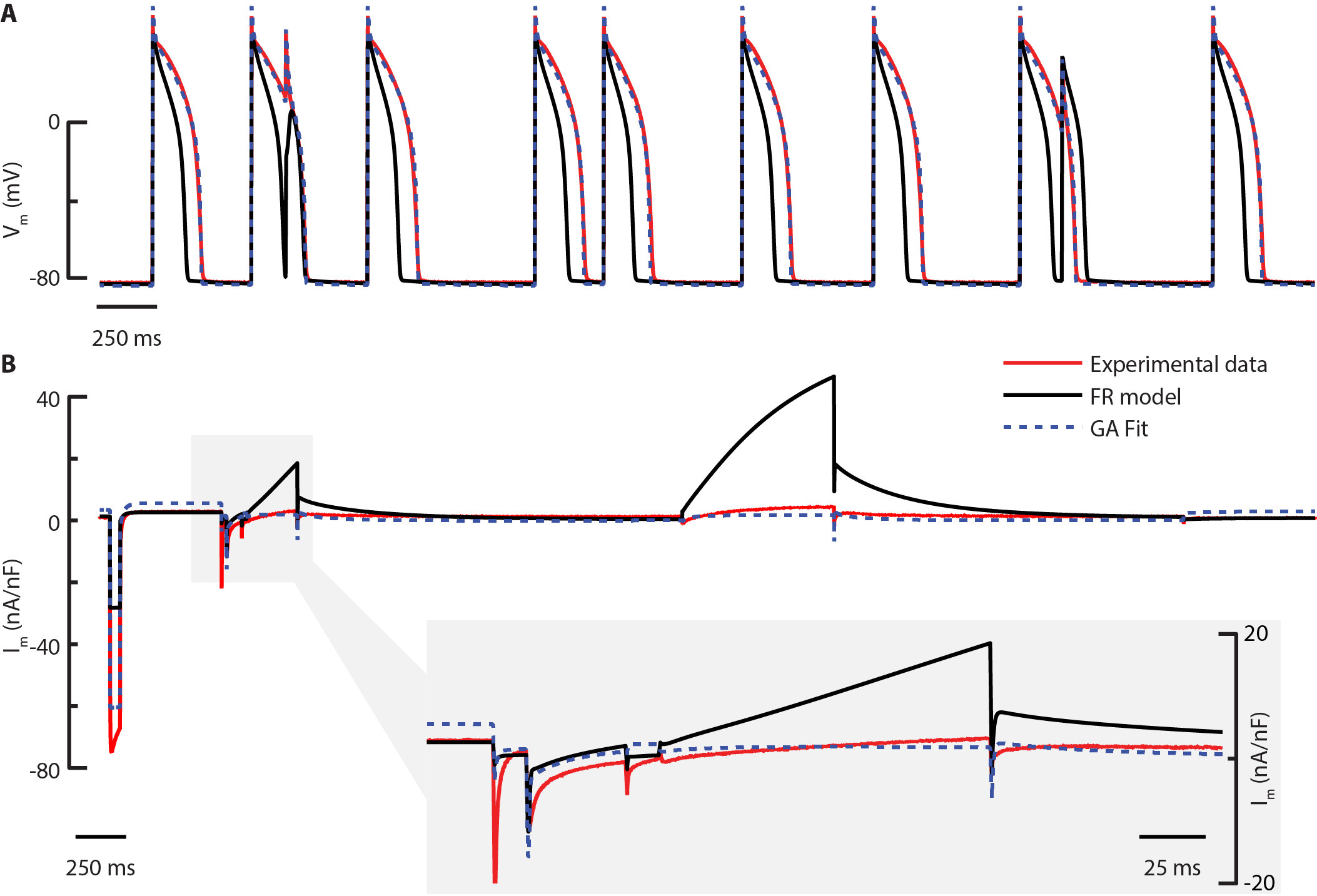

Supplement: S7 Fig — Stochastic pacing and voltage clamp fits of the experimental data of cell 1. The figure shows the best individual from 10 GA runs using the iterative approach (blue), the original FR model (black) and the experimental data (red). The GA fit shows a closer match with the experimental data than the FR model. Stimulus artifacts and capacitative currents were removed (as in Fig 5), but data sets were plotted as continuous traces to ease visualization. (PNG) [file pcbi.1004242.s008.png]

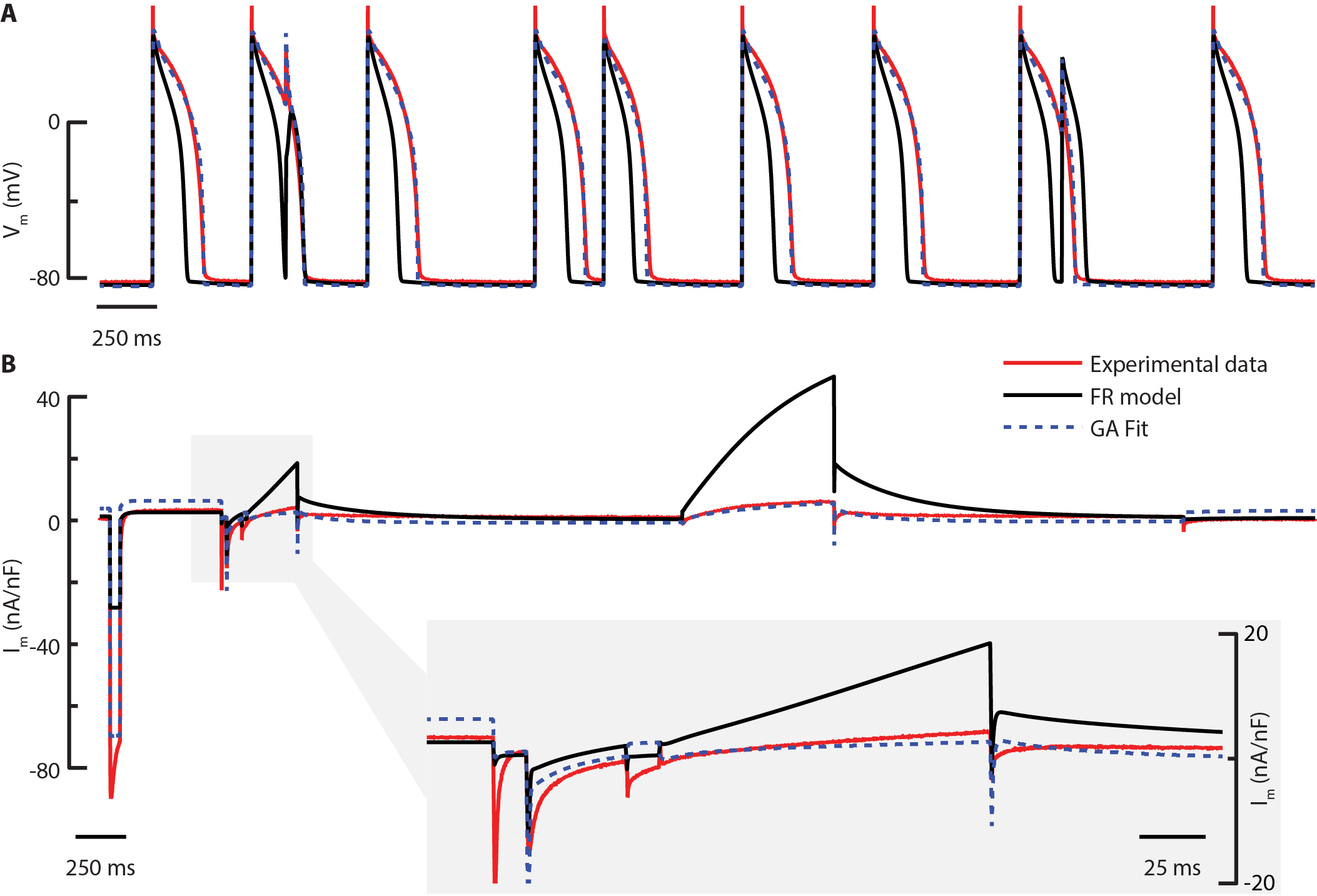

Supplement: S8 Fig — Stochastic pacing and voltage clamp fits (blue) of the experimental data of cell 3 (red) compared to the original FR model (black). (PNG) [file pcbi.1004242.s009.png]

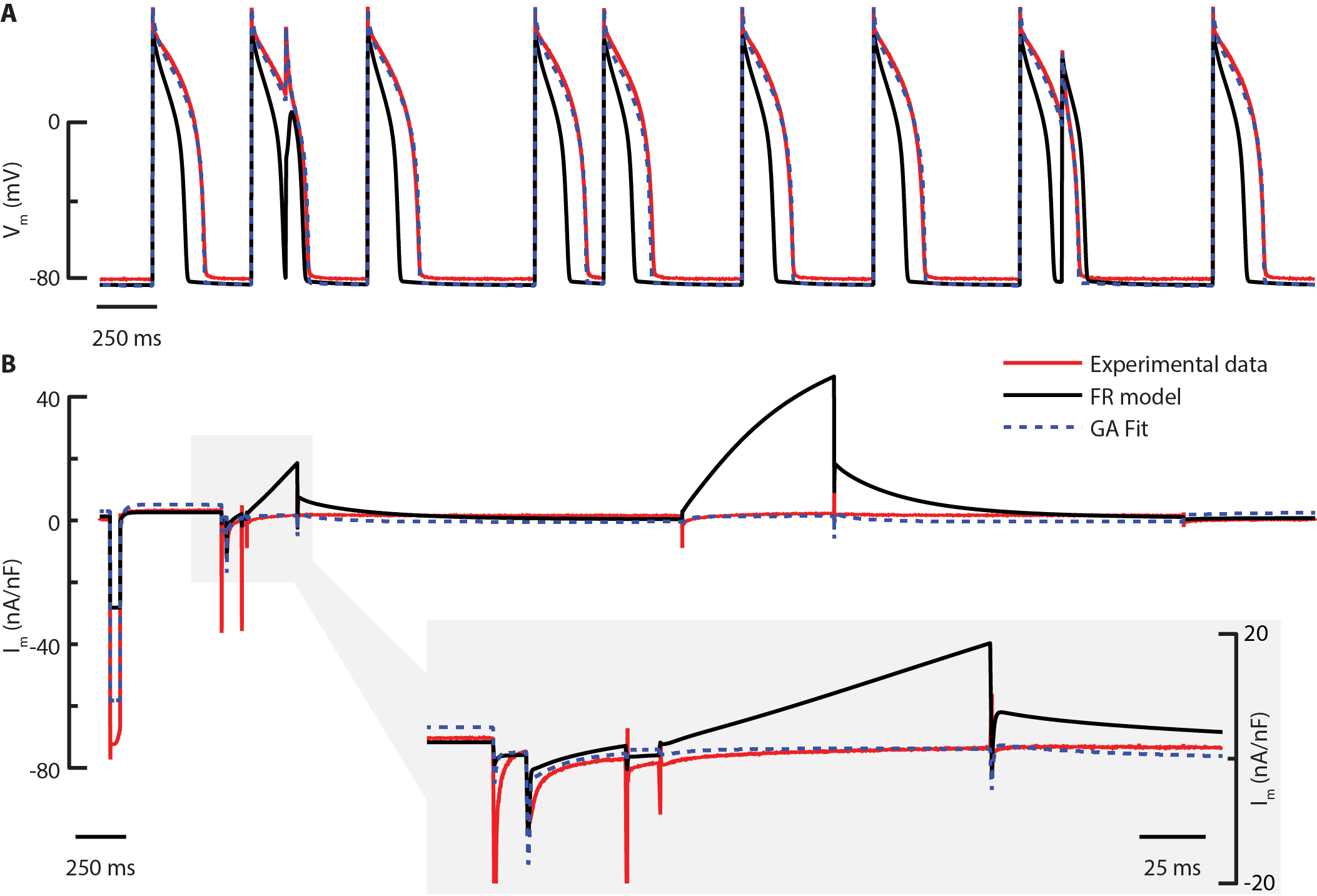

Supplement: S9 Fig — Stochastic pacing and voltage clamp fits (blue) of the experimental data of cell 4 (red) compared to the original FR model (black). (PNG) [file pcbi.1004242.s010.png]
