# Supplementary material for: Cell-Specific Cardiac Electrophysiology Models
Source: PLoS Comput Biol. 2015 Apr 30;11(4):e1004242. doi: 10.1371/journal.pcbi.1004242 (PMC4415772; doi:10.1371/journal.pcbi.1004242)
Supplement: S5 Fig — Parameters were scaled to 80, 90, 95, 105, 110 and 120% of the published value and the sum of squared errors (using intracellular calcium concentration rather than transmembrane potential or total current in Eqs 2 and 3) was calculated and visualized here as the sensitivity. For each parameter, the effect of the scaling is given from small to large parameter scaling, i.e., from 80–120%. The calcium signal is most sensitive to parameters that are directly calcium-related (ICaL, JSERCA, and IpCa). (PDF) [file pcbi.1004242.s006.pdf]

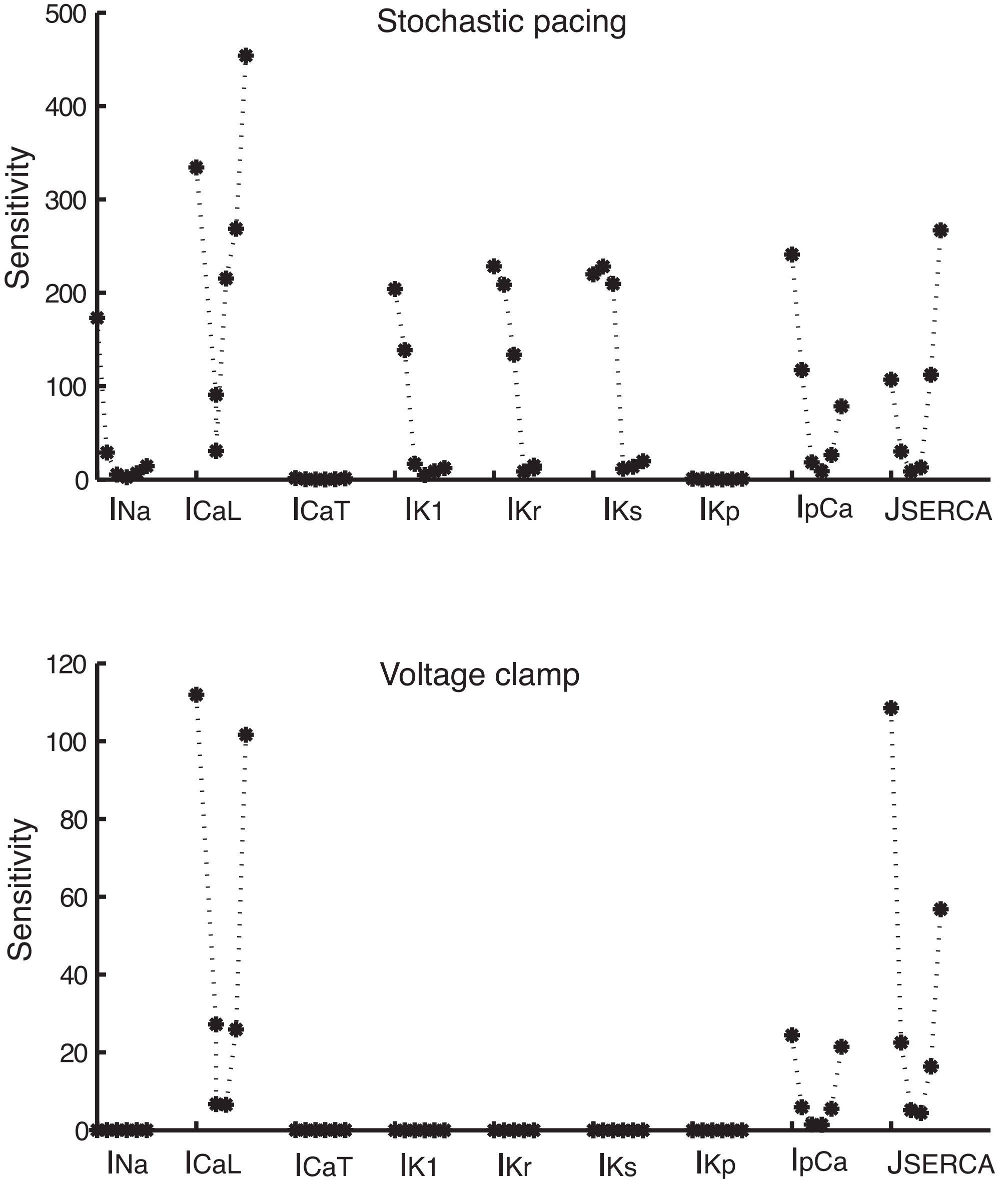

Stochastic pacing
Sensitivity
500
400
300
200
100
0
INa
ICaL
ICaT
IK1
IKr
IKs
IKp
IpCa
JSERCA
Voltage clamp
Sensitivity
120
100
80
60
40
20
0
INa
ICaL
ICaT
IK1
IKr
IKs
IKp
IpCa
JSERCA
